# Quantitative evaluation of the time-course and efficacy of targeted agents for ulcerative colitis

**DOI:** 10.3389/fphar.2024.1399963

**Published:** 2024-06-05

**Authors:** Boran Yu, Siyao Jin, Jiaqi Han, Jiamin Xu, Shaolong Zhang, Yanming Li, Xiangyu Ma, Xiaoling Wang, Libo Zhao

**Affiliations:** ^1^ Department of Pharmacy, Beijing Children’s Hospital, Capital Medical University, National Center for Children’s Health, Beijing, China; ^2^ Department of Pharmacy, Peking University Third Hospital, Beijing, China

**Keywords:** model-based meta-analysis, ulcerative colitis, drug efficacy, biologics, small targeted molecules

## Abstract

**Background:**

Targeted agents are widely utilized in the treatment of ulcerative colitis (UC). Hence, a comprehensive understanding of comparative drug efficacy in UC is of great importance for drug development and clinical practice. Our objective was the quantitative evaluation of the comparative efficacy of targeted agents for UC.

**Methods:**

Three mathematical models were developed based on data from randomized controlled trials in patients with moderate-to-severe UC to describe the time-course and dose-response of efficacy defined as clinical remission, clinical response, and endoscopic improvement, as well as the placebo effect. The covariate effects were further evaluated. Model simulation was performed in a hypothetical population to compare the efficacies across different drugs.

**Results:**

The analysis dataset was composed of data from 35 trials of 12 drugs in UC. Time–response relationships were evaluated that indicated a gradual onset of drug efficacy in adalimumab, ozanimod, and Janus kinase (JAK) inhibitors. The dose-response relationships were estimated for each drug respectively. Patient age, disease duration, baseline weight, prior tumor necrosis factor (TNF) inhibitor exposure, and current treatment with corticosteroid showed an impact on efficacy, suggesting that younger patients with shorter UC duration without prior anti-TNF treatment and current corticosteroids therapy tend to display greater treatment effects.

**Conclusion:**

This study developed three longitudinal models for UC to quantitatively describe the efficacy of targeted agents, as well as the influencing factors of efficacy. Infliximab and upadacitinib were determined to be the most effective biological and small targeted molecules, respectively. These findings may provide valuable implications for guiding future decision-making in clinical practice and drug development for UC.

## Introduction

Ulcerative colitis (UC) is a chronic inflammatory disease that primarily impacts the colonic mucosa (Ungaro et al., 2017). It is estimated that UC affects five million people globally (Le Berre et al., 2023). It may lead to impaired health quality and considerable economic burden (Ordás et al., 2012; Ungaro et al., 2017).

Pharmaceutical therapy for UC involves 5-aminosalicylates (5-ASA), corticosteroids, immunomodulators, and targeted agents (Le Berre et al., 2023). Due to the potentially serious adverse effects and the proportion of patients who become dependent or refractory to 5-ASA and corticosteroids, targeted agents, including biologics and small targeted molecules, have been widely used and strongly recommended in the last decade for induction and/or maintenance remission in moderately-to-severely active UC (Raine et al., 2022). Although various biologics targeting pro-inflammatory cytokines like tumor necrosis factor (TNF) -α, α or β integrins and interleukin, as well as small molecules targeting Janus kinase (JAK) and sphingosine-1-phosphate receptor (S1PR), have demonstrated efficacy in clinical trials in moderate to severe UC, it is necessary to compare their efficacy due to the different pharmacological properties and potency (Burr et al., 2021; Lasa et al., 2022).

Several conventional and network meta-analyses have been published assessing the comparative efficacy and safety of these targeted agents (Singh et al., 2020; Burr et al., 2021; Lasa et al., 2022). However, a major limitation of these studies is the challenge of integrating time-course and placebo effects, which tend to vary across different studies. This limitation arises from the fact that drug efficacy was assessed only at one time point across different trials, thus overlooking the longitude dynamic of efficacy for different compounds. Second, previous meta-analyses assessed the impact of baseline characteristics through subgroup analysis, where patients with different characteristics were divided into separate subgroups for comparison. However, this method failed to quantitatively describe the potential influence of baseline characteristics.

Model-based meta-analysis (MBMA) is a combination of quantitative pharmacology and meta-analysis which allows for the evaluation of the full response profile by establishing a time-course model (Wu et al., 2019; Wen et al., 2022). The longitudinal model is able to summarize treatment efficacy measured at different time points, as well as eliminate the heterogeneity of placebo effects and baseline characteristics across different studies (Chen et al., 2021; He et al., 2021). The impact of patient characteristics can also be quantitatively measured with a covariate model, enabling a more accurate estimation of the true efficacy and facilitating valid comparison across treatments in diverse patient populations.

The objectives of this study were to establish placebo effect and pharmacodynamic models to quantitatively evaluate the efficacy of targeted agents that are either approved or in phase Ⅲ clinical investigation for UC. The MBMA method was utilized to calculate the onset time, placebo effects, maximum efficacy, and other pharmacodynamic parameters to clarify the difference across all treatments and assess the impact of patient characteristics. Three longitudinal models were developed for three commonly reported endpoints in UC clinical trials, including clinical remission, clinical response, and endoscopic improvement.

## Materials and methods

### Data collection and quality assessment

The systematic research of the published literature on clinical trials of biologics and small targeted molecules was carried out from inception until 9 April 2023. The following literature resources were utilized: Medline (via PubMed), EMBASE, Cochrane Library, and ClinicalTrials.gov databases, as well as the abstracts from the Digestive Disease Week (DDW), American College of Gastroenterology, and Congress of European Crohn’s and Colitis Organisation. Search keywords included ulcerative colitis, infliximab, adalimumab, golimumab, vedolizumab, etrolizumab, ustekinumab, tofacitinib, filgotinib, upadacitinib, ozanimod, etrasimod, placebo, and “randomized controlled trial.” The detailed search strategy is listed in the Supplementary Materials. A supplementary search was conducted from the references of the previous publications. The systematic review was conducted following Preferred Reporting Items for Systematic Reviews and Meta-Analyses (PRISMA) guidelines (Moher et al., 2009).

The inclusion criteria were as follows: (i) randomized placebo- or active-controlled trials published in English; (ii) trials on patients who were diagnosed with moderate-to-severe UC and treated with biologics or small targeted molecules; (iii) clinical remission, clinical response, or endoscopic improvement was used as the endpoint. Trials of novel agents in development with only phase Ⅰ/II RCT data or pediatric studies were excluded. Concomitant medications at stable dosages were allowed, including mesalamine, oral steroids, and immunomodulators. The exclusion of any study was solely based on the study design and patient characteristics, instead of any judgment of the study results. For crossover trials, only data from the first period were extracted. The quality of the studies included was assessed using the Cochrane risk of bias tool.

To facilitate the MABA process of Boucher and Bennetts (2016) and Yu et al. (2022), the following relevant data were extracted from citations and online open resources for clinical trial registration platforms: study characteristics (publication year, title, author, trial name, drug, dose, regimen, sample size, and primary endpoint), patient demographic characteristics (age, weight, disease duration, prior, and concomitant medications), as well as efficacy endpoints. If the efficacy endpoints were reported as a graph, data were converted with OriginPro (version 9.6.5.169). The search results were screened, and data were extracted independently by two investigators (BY and SJ). Disagreements were settled by discussion and consensus with a third reviewer (SZ). Before modeling, dose regimens were normalized to pool the efficacy data of the same dosage.

### Model development

After graphical exploration, the longitudinal data representing the percentage of patients who achieved clinical remission, clinical response, and endoscopic improvement were characterized using a hierarchical regression model with the maximum likelihood estimation method. The structure of longitudinal models could be generally described as follows (Eqs 1-3)

$$N_{response,ijt}\;∼\;binomial\left(N_{ij},{P\left(response\right)}_{ijt}\right),$$ (1)

$${P\left(response\right)}_{ijt}=g\left(E_{0}+E_{drug}\right),$$ (2)

$$g=1/(1+\exp\left(-\left(E_{0}+E_{drug}\right)\right).$$ (3)

The endpoint is represented by *response* . *N*_*response*_ represents the count of patients achieving the endpoints at *t*th time in *j*th arm of *i*th trial, which follows a binomial distribution with a probability *P*(*response*)_*ijt*_ and a sample size *N*_*ij*_. The logit translation which was performed to restrict the probability scale to a range of 0–1 is represented by *g*.

For each endpoint, the time dependent placebo effect was modeled using polynomial regression, exponential model, or restricted cubic splines to allow for the non-linear associations. The exponential model of placebo (Eq. (4)) was described by intercept (*B*), asymptote (*A*), and rate constant (*k*_*pbo*_).

$$E_{0}=B+A∙\left(1-e^{{-k}_{pbo}∙time}\right).$$ (4)

*E*_*drug*_ is a function depending on drug, dose, time, fixed-effect model parameters *θ*, and covariates *X*_*ij*_ (Eq. (5)).

$$E_{drug}=f\left(drug,dose,time,\theta ,X_{ij}\right)$$ (5)

$$E_{drug}=\left(1-e^{-k∙time}\right)∙\left(E_{\max}∙{dose}^{\gamma }\right)/\left({dose}^{\gamma }+{ED}_{50}^{\gamma }\right)$$ (6)

$$E_{drug}=\left(1-e^{-k∙time}\right)∙S_{drug}∙dose$$ (7)

An exponential model was used to characterize the time-varying drug efficacy, and a sigmoidal Emax or linear model was used to describe the potential dose–response relationship (Equation 6-7). Throughout the model development process, parameter *k* was estimated separately for each drug or drug category; *k* was estimated to describe the onset of drug efficacy for drugs, with efficacy increased progressively and finally the plateau. For drugs with limited dynamic data, *E*_*drug*_ was estimated without employing an exponential model, indicating a consistent efficacy over observed duration. In the Emax model, *E*_max_ represents the maximum efficacy of each treatment and *ED*_*50*_ denotes the dose required to attain 50% of maximum efficacy. Hill coefficient (*γ*) was set to 1 in our study due to the limited dose–response information available for each drug. The parameter *ED*_*50*_ was fixed to 0 for drugs for which *ED*_*50*_ failed to be estimated as a reasonable value due to the limited dose regimens or the absence of a clear dose–response relationship, indicating a consistent efficacy across different dose regimens. The linear model was also applied to describe the potential linear dose–response relationship of specific drugs.

Multiple levels of heterogeneity were measured as between- and within-study variability. Between-study variability was introduced thus (Eq. 8)

$$P_{i}=P_{pop}+\eta_{ij},$$ (8)

where *P*_*i*_ is the individual value of the parameters, *P*_*pop*_ is the typical value of the parameters, and *η*_*ij*_ is the random residual from between-arm variability. The residual error model (*ε*) was introduced with a weight based on the standard error and the sample size of fitted values (Eq. (9)), where *OBS*_*ijt*_ represents the observed drug efficacy, *P*_*ijt*_ is the predicted value of drug efficacy at *t*th time in *j*th arm of *i*th trial, and *N*_*ij*_ represents the sample size of *j*th treatment of *i*th trial.

$${OBS}_{ijt}=P_{ijt}+\sqrt{P_{ijt}∙\left(1-P_{ijt}\right)/N_{ij}}∙\varepsilon$$ (9)

*P* and *N* were added to the residual error model to avoid the possible deviations caused by extreme efficacy values and suggest that a larger sample size related to less residual error.

To further develop the models, patient population characteristics at baseline were included as covariates to explain variability across trials and arms. Factors potentially affecting drug efficacy were evaluated, including patients’ age, weight, disease duration, site of disease, percentage of males, baseline disease activity, and prior and concomitant medications. Summary-level patient characteristics were modeled thus (Eqs 10, 11)

$$P_{i}=P_{pop}+\left({COV}_{ij}\;-\;{COV}_{mean}\right)∙\theta_{COV}$$ (10)

$$P_{i}=P_{pop}\times {\left({COV}_{ij}\;/\;{COV}_{mean}\right)}^{\theta_{COV}}$$ (11)

*P*_*i*_ is the parameter value of *j*th arm in the *i*th trial. *P*_*pop*_ is the typical parameter value of the placebo or treatment. *COV*_*ij*_ is the covariate value of *j*th arm in *i*th trial. *COV*_*mean*_ is the mean covariate value of the overall placebo and treatment groups. *θ*_*COV*_ is the parameter describing the relationship between covariates and efficacy parameters. The covariates were added to the model in a stepwise way, with only clinically and statistically reasonable covariates included in the final model. Finally, the within-arm autocorrelation structure was included and tested in a different form, including AR1, AR2, compound symmetry, and autoregressive moving average structure. The development of the final model was based on the Akaike information criterion and the log likelihood ratio with an acceptance *p*-value of 0.05.

### Model validation and simulation

The model’s adequacy was evaluated by conducting model diagnostic plots, including a comparison of the conditional weight residual across the time course and with population-predicted values. A visual prediction check (VPC) was developed to evaluate the final model predictive performance. A total of 1,000 simulations of the final model were conducted, and performance was confirmed according to the consistency between the 95% confidence interval (CI) and the observed values. The stability of the final models was also assessed by the bootstrap method with 1,000 times repeating sampling and re-estimating of the parameters. The median bootstrap parameter values were compared with the respective values of the model estimation.

Based on the parameter estimation, model simulation was performed to generate the efficacy of included targeted agents in a hypothetical population throughout the entire follow-up duration. The drug response at week 12 was compared by predicted typical value and its 95%CI. The efficacy simulations were conducted using Monte Carlo simulation of 10,000 times.

Quality assessment of the studies was performed with Review Manager, version 5.4 (Cochrane Training). Model development, validation, and simulation were carried out with R software, version 4.3.1 (R Foundation for Statistical Computing) and R Studio, version 2023.06.1 (Posit Software). The relevant code can be found in the Supplementary Materials.

## Results

### Database overview

After a systematic review, 35 trials containing 95 treatment arms and 15,585 patients (mean age: 41.2 years; 59.3% male; diagnosed with UC for 7.1 years) were selected and included in the analysis from a pool of 11,190 retrieved citations. Of these, clinical remission was reported in 30 trials, and clinical response and endoscopic improvement were reported in 34 trials. A flow diagram (Supplementary Figure S1) illustrates the process of study selection. The list of included trials and reported time points is available in Supplementary Table S1. The result of quality assessment suggested that the trials included were of relatively high quality with a low risk of bias; detailed information is available in the Supplementary Materials.

A total of seven biologics and five small molecules were involved in this study, including TNF -α inhibitors, integrin inhibitors, interleukin inhibitors, JAK inhibitors, and S1PR modulators. Comprehensive details regarding the drug classification and baseline characteristics can be found in Table 1.

**TABLE 1:** Summary of the treatments and baseline characteristics of the included studies.

Drug	Trials	Patients	Arms	Percentage of male individuals (%)^a^	Age (years)^a^	Weight (kg)^a^	Disease duration (years)^a^	Prior anti-TNF therapy (%)^a^	Concomitant corticosteroid (%)^a^
TNF-α inhibitor									
Infliximab	7	996	10	61.4 (42.0, 66.7)	39.8 (34.1, 42.4)	70.0 (57.6, 80.0)	5.9 (3.7, 8.4)	0.0 (0.0, 0.0)	54.4 (43.5, 80.0)
Adalimumab	7	2,237	10	57.8 (56.0, 67.8)	40.5 (39.6, 44.4)	75.4 (73.4, 76.8)	7.8 (6.4, 8.3)	0.0 (0.0, 39.1)	55.8 (36.3, 72.4)
Golimumab	1	690	3	55.6 (54.4, 60.7)	40.7 (40.0, 40.9)	NA	6.4 (6.4, 6.6)	0.0 (0.0, 0.0)	43.8 (42.9, 48.6)
Integrin inhibitor									
Vedolizumab	3	772	3	60.8 (58.7, 67.1)	40.8 (40.1, 44.0)	72.4 (58.6, 72.7)	7.3 (6.1, 8.6)	42.2 (20.8, 50.0)	36.1 (30.8, 56.0)
Etrolizumab	5	948	6	59.0 (51.0, 68.0)	40.5 (40.0, 44.4)	81.8 (74.8, 88.8)	8.6 (8.0, 9.2)	30.5 (0.0, 100.0)	43.6 (41.0, 47.0)
Interleukin inhibitor									
Ustekinumab	1	642	2	60.0 (59.4, 60.6)	42.0 (41.7, 42.2)	73.4 (73.0, 73.7)	8.2 (8.1, 8.2)	68.4 (67.8, 68.9)	53.2 (52.2, 54.1)
Mirikizumab	2	1,054	4	61.0 (59.7, 62.8)	42.6 (41.8, 43.4)	75.6 (73.0, 77.0)	8.2 (6.0, 9.0)	37.4 (37.4, 37.4)	46.0 (40.3, 55.7)
JAK inhibitor									
Tofacitinib	3	1,073	8	57.2 (50.0, 64.0)	41.4 (41.0, 43.8)	74.2 (72.9, 75.9)	8.9 (7.6, 10.9)	42.2 (29.0, 55.2)	45.3 (27.0, 58.0)
Upadacitinib	3	864	6	61.6 (48.9, 66.1)	42.1 (37.0, 47.0)	70.3 (69.3, 71.2)	7.1 (6.6, 7.6)	77.2 (69.6, 100.0)	49.1 (38.9, 55.4)
Filgotinib	2	1,069	4	56.6 (50.2, 65.3)	42.5 (42.0, 43.0)	NA	8.5 (6.7, 9.8)	46.6 (0.0, 93.3)	30.1 (22.0, 36.1)
S1PR modulator									
Ozanimod	2	561	3	57.1 (49.2, 71.6)	41.4 (38.8, 41.8)	74.9 (72.3, 77.4)	6.7 (5.9, 6.9)	22.4 (18.5, 30.3)	34.0 (27.7, 40.0)
Etrasimod	3	598	4	55.4 (52.6, 57.7)	40.8 (40.3, 43.2)	NA	6.6 (6.2, 7.0)	31.4 (28.8, 34.0)	30.5 (25.0, 36.0)
**Placebo**	32	4,081	32	60.0 (42.0, 72.9)	41.2 (34.5, 44.8)	72.8 (57.2, 78.7)	7.1 (3.7, 10.2)	30.6 (0.0, 100.0)	47.0 (8.0, 67.6)
**Total**	35	15,585	95	59.3 (23.0, 72.9)	41.2 (34.1, 47.0)	73.3 (57.2, 88.8)	7.1 (3.7, 10.9)	22.4 (0.0, 100.0)	46.0 (7.0, 80.0)

NA, not available.

^a^ Data were reported as median (range).

Before modeling, the missing values of covariates were imputed with the mean values of corresponding available data after a thorough examination of the database. The dose regimens were reviewed, and the dosage of ustekinumab was normalized based on the assumption of a typical patient weight of 70 kg.

### Model development

#### Clinical remission model

The placebo effect in the clinical remission model was estimated using an exponential model with the intercept estimated as −39.26 (95CI%: −142.73–64.22), asymptote estimated as 37.04 (95CI%: −66.38–140.45), and rate constant estimated as 0.66 (95%CI: 0.34–1.27), indicating that the ET_90_ (when 90% of maximal efficacy was achieved) for placebo was 8.9 weeks. Parameter *k* was estimated for adalimumab, filgotinib, and ozanimod to describe the time-varying drug efficacy. According to the model, ET_90_ was estimated as 12.1, 47.1, and 23.1 weeks for adalimumab, filgotinib, and ozanimod, respectively. However, for other drugs, the inclusion of parameter *k* did not result in an improvement of the model fit. For these drugs, the onset of drug efficacy aligns with the trend of the placebo effect. The maximum efficacy of each drug was estimated individually with a fixed parameter *E*_max_ as no obvious dose–response association was identified throughout the model development. When testing covariates, disease duration normalized to the mean value of 7.53 years, significantly affecting the asymptote in placebo effect and *E*_max_ of drugs. The estimated parameter values of the final models are shown in Table 2.

**TABLE 2:** Parameter estimations in final models and bootstrap results.

Parameter	Clinical remission		Clinical response		Endoscopic improvement	
	Estimate (95 CI%)	Bootstrap median (95 CI%)	Estimate (95 CI%)	Bootstrap median (95 CI%)	Estimate (95 CI%)	Bootstrap median (95 CI%)
**Dose–response model**						
Infliximab	1.48 (1.28, 1.67)^a^	1.47 (1.34, 1.60)^a^	1.03 (0.852, 1.22)^a^	1.03 (0.789, 1.29)^a^	1.22 (1.07, 1.37)^a^	1.21 (0.991, 1.42)^a^
Adalimumab	0.864 (0.609, 1.12)^a^	0.842 (0.740, 0.954)^a^	0.704 (0.618, 0.790)^a^	0.720 (0.581, 0.854)^a^	0.586 (0.453, 0.718)^a^	0.580 (0.376, 0.805)^a^
Golimumab	1.35 (0.930, 1.78)^a^	1.36 (1.24, 1.46)^a^	0.616 (0.425, 0.807)^a^	0.618 (0.471, 0.856)^a^	1.12 (0.880, 1.37)^a^	1.12 (0.965, 1.27)^a^
Vedolizumab	1.24 (1.01, 1.47)^a^	1.22 (1.09, 1.35)^a^	1.15 (1.01, 1.29)^a^	1.17 (0.989, 1.39)^a^	1.18 (0.947, 1.40)^a^	1.18 (1.06, 1.32)^a^
Etrolizumab	0.859 (0.629, 1.09)^a^	0.846 (0.757, 0.939)^a^	0.661 (0.477, 0.845)^a^	0.639 (0.433, 0.801)^a^	0.715 (0.529, 0.900)^a^	0.699 (0.453, 0.945)^a^
Ustekinumab	0.864 (0.564, 1.16)^a^	0.869 (0.772, 0.968)^a^	1.30 (1.01, 1.59)^a^	1.34 (1.00, 1.70)^a^	0.751 (0.463, 1.04)^a^	0.769 (0.632, 0.933)^a^
Mirikizumab	1.06 (0.832, 1.29)^a^	1.02 (0.711, 1.13)^a^	1.27 (1.07, 1.46)^a^	1.13 (0.504, 1.43)^a^	0.763 (0.559, 0.967)^a^	0.609 (−0.548, 0.931)^a^
Tofacitinib	1.15 (0.911, 1.39)^a^	1.18 (1.00, 1.47)^a^	1.88 (1.28, 2.47)^a^	2.11 (1.15, 4.64)^a^	0.732 (0.508, 0.957)^a^	0.898 (0.620, 2.05)^a^
Filgotinib	1.54 (1.20, 1.88)^a^	1.66 (1.08, 2.80)^a^	6.87 × 10^−3^ (5.09 × 10^−3^, 8.66 × 10^−3^)^b^	7.28 × 10^−3^ (4.02 × 10^−3^, 1.19 × 10^−2^)^b^	0.721 (0.551, 0.892)^a^	0.719 (0.227, 1.31)^a^
Upadacitinib^c^	1.35 (1.10, 1.60)^a^	1.33 (1.02, 1.54)^a^	0.234 (−0.762, 1.23)^a^	0.241 (−0.976, 1.36)^a^	3.18 × 10^−2^ (2.52 × 10^−2^, 3.74 × 10^−2^)^b^	3.14 × 10^−2^ (2.22 × 10^−2^, 4.19 × 10^−2^)^b^
Upadacitinib 45 mg	1.35 (1.10, 1.60)^a^	1.33 (1.02, 1.54)^a^	2.90 (2.004, 3.79)^a^	3.16 (1.64, 7.12)^a^	3.18 × 10^−2^ (2.52 × 10^−2^, 3.74 × 10^−2^)^b^	3.14 × 10^−2^ (2.22 × 10^−2^, 4.19 × 10^−2^)^b^
Ozanimod	0.934 (0.153, 1.71)^a^	0.947 (0.756, 1.38)^a^	0.428 (0.263, 0.594)^a^	0.426 (0.241, 0.743)^a^	0.348 (0.0946, 0.601)^a^	0.363 (0.265, 0.529)^a^
Etrasimod	NA	NA	0.544 (0.442, 0.646)^b^	0.527 (0.259, 0.627)^b^	0.442 (0.333, 0.552)^b^	0.431 (0.287, 0.597)^b^
**Time-course model**						
*k*_*adalimumab*_	0.198 (7.66 × 10^−2^, 0.511)	0.220 (0.142, 0.361)	NA	NA	NA	NA
*k*_*filgotinib*_	4.76 × 10^−2^ (2.42 × 10^−2^, 9.36 × 10^−2^)	4.44 × 10^−2^ (1.32 × 10^−2^, 9.30 × 10^−2^)	NA	NA	NA	NA
*k*_*ozanimod*_	9.78 × 10^−2^ (6.99 × 10^−3^, 1.37)	9.99 × 10^−2^ (3.28 × 10^−2^, 0.209)	NA	NA	NA	NA
*k*_*JAK*_ _*inhibitor*_	NA	NA	0.103 (6.79 × 10^−2^, 0.157)	0.0973 (3.34 × 10^−2^, 0.247)	NA	NA
**Covariate**						
*COV*_*duration*_ (on *E*_*drug*_)	0.589 (−4.55 × 10^−2^, 1.22)^d^	0.583 (9.21 × 10^−2^, 1.14)^d^	−0.829 (−1.26, −0.395)^d^	−0.928 (−1.63, −0.262)^d^	−5.38 × 10^−4^ (−7.20 × 10^−4^, −3.55 × 10^−4^)^e^	−5.78 × 10^−4^ (−9.41 × 10^−4^, −2.96 × 10^−4^)^e^
*COV*_*duration*_ (on *E*_*0*_)	0.695 (0.385, 1.01)^d^	0.696 (0.474, 0.927)^d^	NA	NA	−1.24 (−1.60, −0.873)^d^	−1.17 (−1.70, −0.460)^d^
*COV*_*TNF*_ (on *E*_*drug*_)	NA	NA	NA	NA	−2.85 × 10^−2^ (−3.82 × 10^−2^, −1.88 × 10^−2^)^e^	−2.71 × 10^−2^ (−4.32 × 10^−2^, −1.10 × 10^−2^)^e^
*COV*_*TNF*_ (on *E*_*0*_)	NA	NA	−2.90 × 10^−3^ (−4.65 × 10^−3^, −1.16 × 10^−3^)^e^	−3.04 × 10^−3^ (−5.17 × 10^−3^, −9.21 × 10^−4^)^e^	NA	NA
*COV*_*age*_ (on *E*_*drug*_)	NA	NA	−2.31 × 10^−3^ (−5.17 × 10^−3^, 5.53 × 10^−4^)^e^	−2.34 × 10^−3^ (−5.65 × 10^−3^, 1.18 × 10^−3^)^e^	NA	NA
*COV*_*corticosteroid*_ (on *E*_*drug*_)	NA	NA	−0.759 (−1.07, −0.444)^d^	−0.857 (−1.42, −0.335)^d^	NA	NA

Abbreviations: CI, confidence interval; NA, not available; *k*_*adalimumab*_, rate constant for the onset of adalimumab; *k*_*filgotinib*_, rate constant for the onset of figotinib; *k*_*ozanimod*_, rate constant for the onset of ozanimod; *k*_*JAK, inhibitor*_, rate constant for the onset of Janus kinase inhibitors; *COV*_*duration*_, covariate parameter of disease duration; *COV*_*TNF*_, covariate parameter of prior tumor necrosis factor inhibitor treatment; *COV*_*age*_, covariate parameter of patients’ age; *COV*_*corticosteroid*_, covariate parameter of concomitant corticosteroid treatment; *E*_*drug*_, drug pure efficacy; *E*_*0*_, placebo effect.

^c^ Upadacitinib with dose regimen of 7.5/15/30 mg once daily.

^a^ Estimation of parameter *E*_max_. The maximum effect is estimated with a consistence parameter E_max_ across all dose regimens.

^b^ Estimation of parameter *slope*. The maximum effect is estimated with a linear dose–response relationship (i.e., the E_max_ of any dose of etrasimod = etrasimod (*slope*) * dose).

^d^ Covariate parameters with a power function model.

^e^ Covariate parameters with a proportional model.

#### Clinical response model

Restricted cubic splines were utilized to effectively characterize the non-linear placebo effect in the clinical response model. An exponential model was formulated to characterize the time course of efficacy for JAK inhibitors (Table 1), and the ET_90_ of the JAK inhibitor was estimated as 22.4 weeks. A linear model was employed to describe the relationship between dosage and efficacy in filgotinib and etrasimod. Attempts were made to describe their dose-response relationships of the other drugs using the Emax model. However, the estimation of *ED*_*50*_ resulted in a model of poor accuracy. Consequently, the *ED*_*50*_ was subsequently fixed at 0, and the drug efficacies were characterized using only the parameter *E*_max_. Notably, the efficacy of upadacitinib at a dosage of 45 mg once daily was higher than other dosage regimens, thus, the *E*_max_ was estimated separately for the specific dosage. Disease duration, patient age, the proportion of patients with prior anti-TNF treatment, and concomitant corticosteroid use were incorporated into the model as covariates. The estimated parameters revealed that the population with younger patients, shorter duration of UC, and lower proportion of prior anti-TNF and current corticosteroid exposure tended to show greater treatment effects.

#### Endoscopic improvement model

Like the clinical response model, a restricted cubic splines method was used to estimate the placebo effect in the endoscopic improvement model. Due to the limited time points available, no time course of drug efficacy was included in the final model. During the exploration of the dose–response relationship, a linear model was estimated for upadacitinib and etrasimod. In the process of covariate screening, the duration of UC and proportion of patients with prior anti-TNF treatment were included in the final model. The estimated parameters indicated that TNF-inhibitor-naïve patients with longer disease duration were expected to have more endoscopic improvement.

### Model evaluation

**FIGURE 1:**
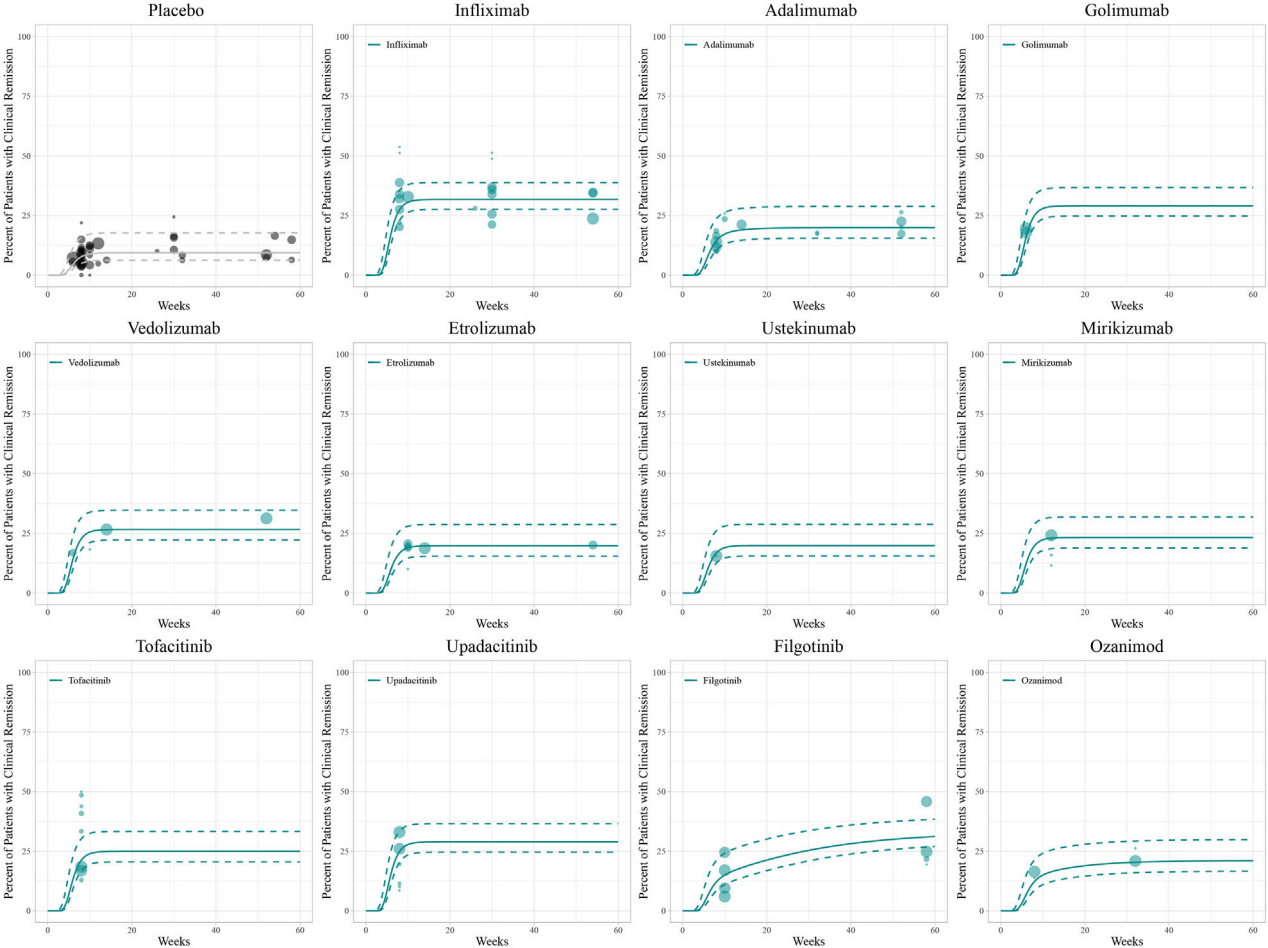
Model fitted time-course plots of the percentage of patients with clinical remission. Solid points represent observed efficacy data, and symbol size represents the sample size. Lines are the model-predicted 2.5th, 50th, and 97.5th percentiles of drug efficacy.

**FIGURE 2:**
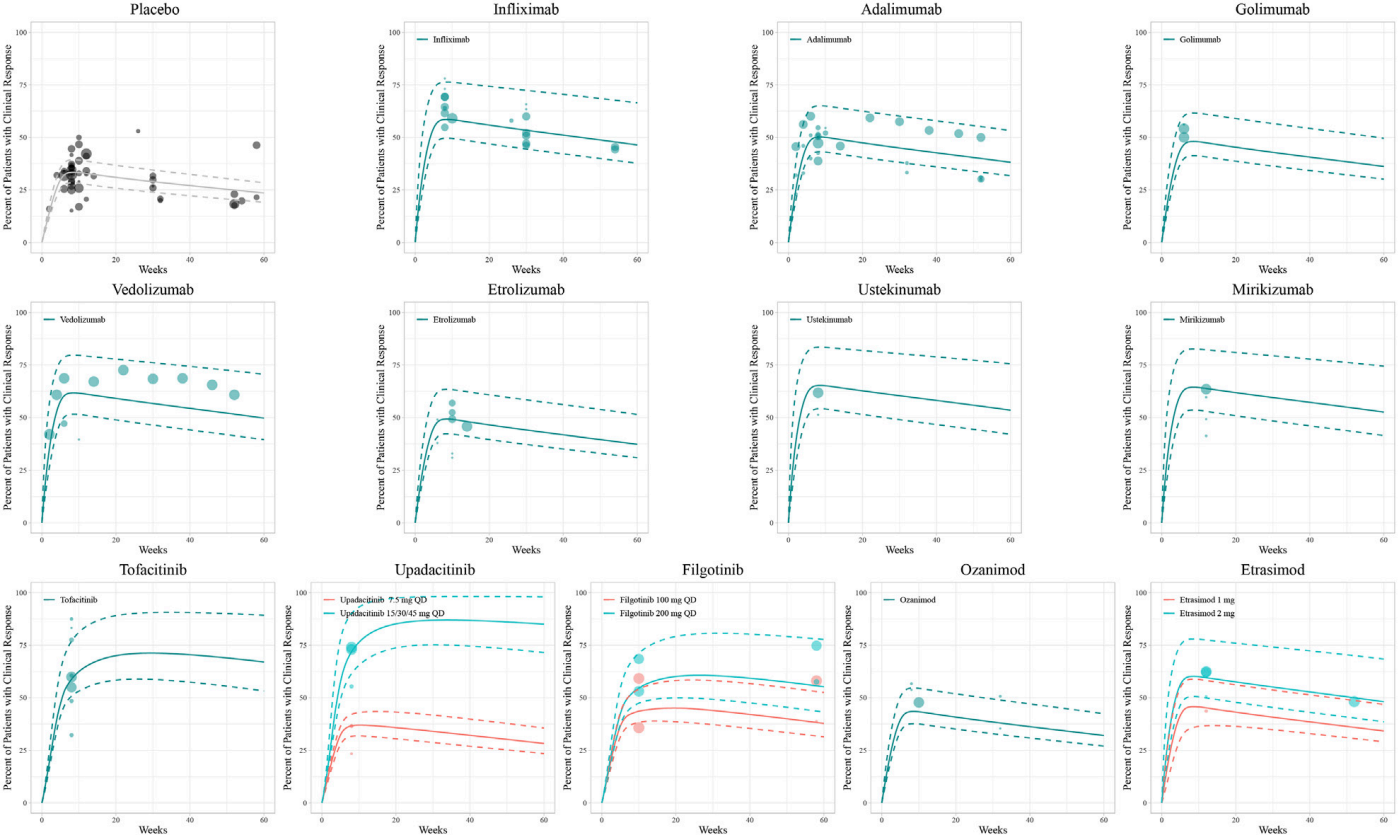
Model fitted time-course plots of the percentage of patients with clinical response. Solid points represent observed efficacy data, symbol size represents the sample size, and different colors represent different dose regimens. Lines are the model-predicted 2.5th, 50th, and 97.5th percentiles of drug efficacy.

**FIGURE 3:**
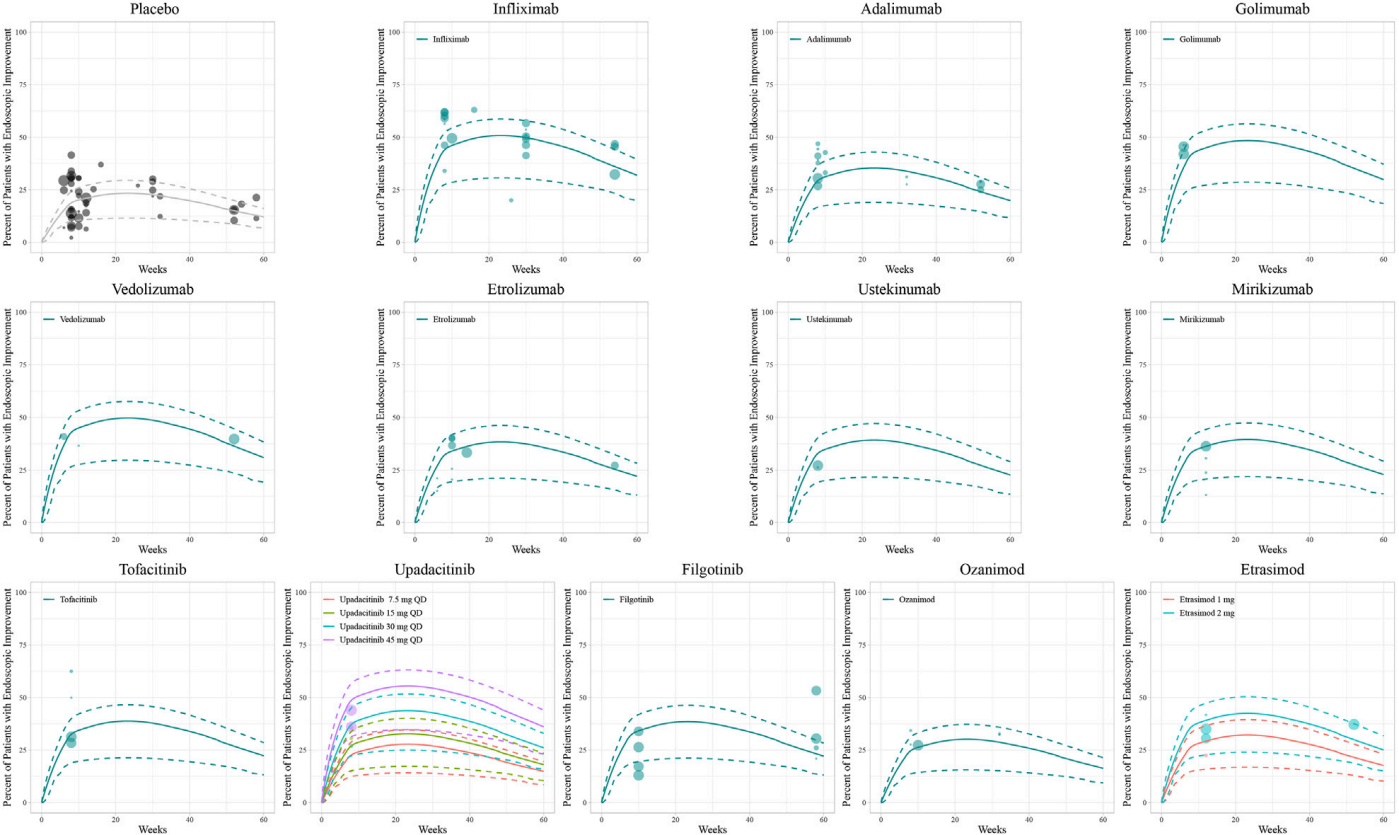
Model fitted time-course plots of the percentage of patients with endoscopic improvement. Solid points represent observed efficacy data, symbol size represents the sample size, and different colors represent different dose regimens. Lines are the model-predicted 2.5th, 50th, and 97.5th percentiles of drug efficacy.

The diagnostic plots of the model demonstrated good predictive performance of the final models, showing no systematic deviations (Supplementary Figure S4). Model-fitted time-course plots of each trial were also conducted (Supplementary Figure S5). In VPC, the temporal dynamics of all treatments were effectively represented, with most of the observed values within the 95% CI of predicted values, illustrating the predictive performance of final models (Figures 1–3). To assess the robustness of the final models, 1,000-times repeated bootstraps were performed, of which 991, 998, and 848 iterations were successful in clinical remission, clinical response, and endoscopic improvement model, respectively. The parameter estimates based on the bootstraps were close to those derived from the original dataset.

### Model simulation

Based on the final models, the typical efficacy of treatments as measured in clinical remission, clinical response, and endoscopic improvement were simulated in a hypothetical population (UC disease duration of 7 years, age 40 years, 50% having received prior TNF-α inhibitor treatment, and 50% currently using corticosteroids) over 60 weeks, and at week 12 (evaluating the efficacy of inducing remission) along with a 95% CI.

**FIGURE 4:**
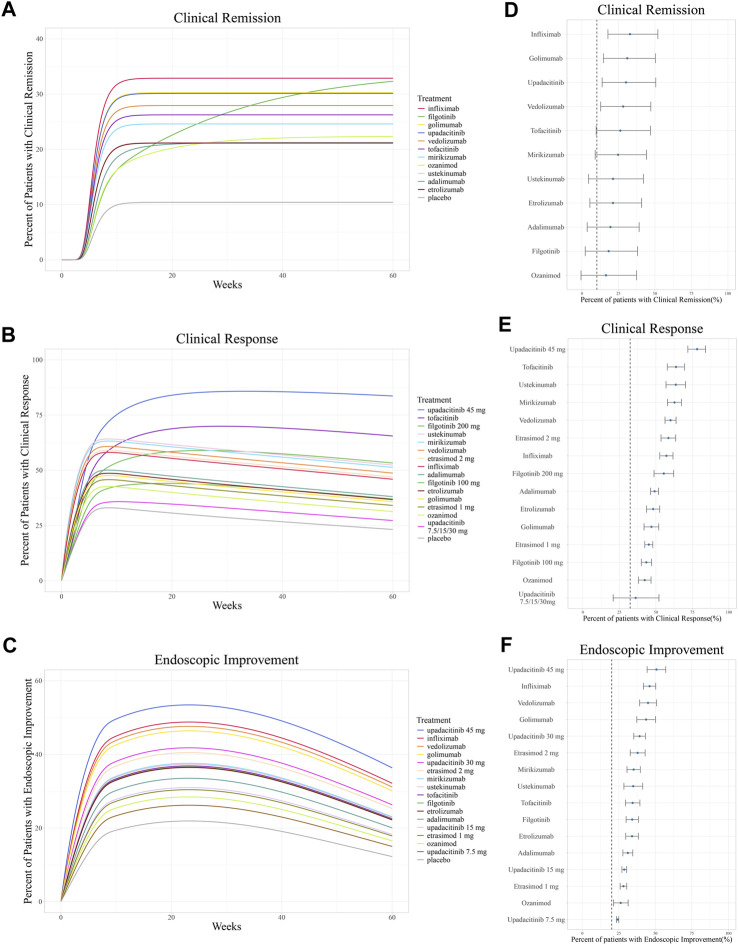
Ranking of treatments by the predicted placebo-corrected median percent of patients with **(A, B)** clinical remission, **(C, D)** clinical response, and **(E, F)** endoscopic improvement across the treatment course and at week 12 (from high to low). Point estimates and 95% confidence intervals predicted from a model simulation of N = 10,000. Dashed lines represent simulated placebo efficacy. For treatments with multiple dosage regimens, only regimens with different efficacy are listed separately.

For clinical remission, the onset of placebo and most drugs’ responses occurred rapidly with a T_90_ of 8.9 weeks. However, a gradual onset of efficacy was particularly demonstrated in adalimumab, ozanimod, and filgotinib (Figure 4A). The simulation of clinical remission at 12 weeks is shown in Figure 4D, revealing that infliximab (33.1%, 95% CI: 18.6%–51.4%) had the best response in clinical remission, followed by golimumab (31.7%, 95% CI: 15.9%–50.0%) and upadacitinib (30.1%, 95% CI: 14.4%–49.8%).

In the simulation of the clinical response model, a trend of losing response was observed in biologics and S1PR modulators after reaching maximum efficacy at approximately 8 weeks (Figure 4B). In contrast, JAK inhibitors exhibited a more gradual and sustained clinical response. At week 12, upadacitinib 45 mg once daily (78.2%, 95% CI: 71.9%& to 84.0%) ranked first in inducing a clinical response, followed by tofacitinib (63.8%, 95% CI: 57.8%–69.5%). Ustekinumab (63.6%, 95% CI: 56.9%–70.2%) was the most effective biologic agent measured in terms of clinical response (Figure 4E).

The endoscopic improvement simulation over 60 weeks and at week 12 is shown in Figure 4C,F. Upadacitinib 45 mg once daily (50.8%, 95% CI: 44.4%–57.2% at week 12) was observed to be the most effective treatment in inducing endoscopic improvement, followed by infliximab (46.1%, 95% CI: 41.8%–50.3% at week 12).

## Discussion

Our study provided quantitative information in comparing the efficacy across 12 targeted agents in moderate-to-severe UC using the MBMA principle. Three independent hierarchical models were developed using publicly available clinical trial data to comprehensively characterize the time-varying drug efficacy, as measured in clinical remission, clinical response, and endoscopic improvement. These three endpoints were the most used end points in clinical trials, representing various levels of improvement in UC. This study included clinical remission defined on either Mayo score or partial Mayo score (which includes the full Mayo score components except for the endoscopic score), as their consistency has been demonstrated (Lewis et al., 2008) and endoscopic improvement has been modeled separately. Clinicians could choose their favorable treatment depending on their priorities.

To make better use of the longitudinal data, the time-course model was incorporated into the final models. The functional form of the placebo effect in the clinical remission model, which describes a gradual increase to a maximal level (at week 8.9) and maintains stability, is consistent with the time course of drug efficacy defined in previous MBMAs (Wu et al., 2019; Chen et al., 2021). On the contrary, the onset time of the placebo effect in clinical response was significantly faster than clinical remission, which reached its maximum efficacy after approximately 6 weeks. The results suggest that the evaluation of drug efficacy in UC should be no earlier than 10 weeks, as the treatment efficacy may require a certain period to stabilize. Previous comparison of treatments in UC also included studies with a minimum follow-up duration of 6 weeks (Burr et al., 2021). It is worth noting that the simulation in both clinical response and endoscopic improvement showed a similar trend of gradual onset followed by a gradual decline in response. It is reported that about 50% of patients after an initial clinical response stopped biologic therapy due to secondary loss of response (SLR) (Papamichael et al., 2015; Papamichael et al., 2019). The inconsistency between clinical remission and endoscopic improvement has been confirmed in the consensus of the International Organization for the Study of IBD that significant mucosal inflammation can be noticed during complete clinical remission (Turner et al., 2021). The time-course of placebo effects demonstrated the necessity of continuous monitoring of the clinical or endoscopic activity of the disease throughout the management of UC.

This study also found the varying time course across different treatments. For instance, biologics exhibited a rapid onset in inducing clinical response followed by the emergence of SLR; however, a more gradual but stable efficacy was observed in JAK inhibitors. The loss of response was considered one of the intrinsic limitations of biologics, which is associated with their immunogenicity and pharmacokinetic features (Papamichael et al., 2019; Lasa et al., 2022). Small targeted molecules were developed and added to the therapeutic armamentarium to attempt to overcome the aforementioned limitations (Nielsen et al., 2023) and are considered to achieve similar effectiveness as biologics (Singh et al., 2020; Alsoud et al., 2021). However, the safety of tofacitinib (a pan-JAK inhibitor) has raised concerns, particularly regarding the potential risk of venous thromboembolism occurrence. The safety assessment results of tofacitinib across different comparative studies were contentious (Olivera et al., 2020; Lasa et al., 2022); thus, more real-world studies are needed to assess the risks of venous thromboembolism and potential preventive measures (Viola et al., 2023). Moreover, selective JAK-1 inhibitors (upadacitinib and filgotinib included in this study) were investigated with the aim of enhancing the risk-benefit profile within the JAK inhibitors (Danese et al., 2019). Our results suggest that, in the choice of treatment for UC, a comprehensive evaluation of drug characteristics is required.

In summary, the results for our three outcome measures align relatively consistently with the ranking of drug effectiveness in previous studies. TNF-α and JAK inhibitors were the most effective biologic agent and small targeted molecule, respectively. Infliximab ranked first in inducing clinical remission, and upadacitinib 45 mg once daily revealed best efficacy in inducing clinical response and endoscopic improvement. These results were consistent with previously published meta-analyses (Singh et al., 2020; Burr et al., 2021; Lasa et al., 2022). However, partial differences were observed between our results and previous studies, and these variations may be attributable to the difference in the characteristics of the selected population and the other trials in our study.

In this study, disease duration was included in all three models as a covariate on *E*_max_, indicating a significant influence of disease duration on drug efficacy. The results demonstrated better drug efficacy in patients with shorter disease duration. Unlike Crohn’s disease, where biologics are recommended as first-line treatment, there is less evidence in UC supporting early treatment escalation (Raine et al., 2022). This study suggests that the application of biologics or small targeted molecules early in the UC disease course may lead to improved clinical outcomes.

The proportion of patients exposed to anti-TNF agents was also included as a covariate, suggesting reduced efficacy in patients with previous anti-TNF treatment. The result was supported by a previous meta-analysis and may be attributed to the pharmacokinetic factors that led to rapid drug clearance and immunogenicity across drugs (Singh et al., 2018; Vande Casteele et al., 2022; Papamichael et al., 2023). Further studies are expected to explore the predictors of primary non-response and SLR to prevent or delay the necessity of switching to a second-line biologic treatment (Singh et al., 2018; Gisbert and Chaparro, 2020). Moreover, strategies have been proposed to optimize the efficacy of second-line biologics (Gisbert and Chaparro, 2020). For patients experiencing SLR after first anti-TNF therapy because of the formation of antibodies, thiopurine is recommended as a concomitant therapy to prevent the treatment failure with the second anti-TNF agent (Roblin et al., 2020).

There are several strengths to our study. First, the largest number of trials and patients were included in this research, and the latest developed mirikizumab, not included in previous network meta-analyses, was also included. Second, the inclusion of double-blind randomized trials provided low-risk evidence for the quantitative evaluation of drug efficacy. Third, time-course and dose-response models were introduced for a comprehensive description for drug efficacy, enabling a full utilization of different dose regimens over the follow-up period.

There are still some limitations to our analysis. First, the efficacy data along with the time course were not adequate for some drugs, which led to an imprecise result when estimating the time-course and dose–response relationship for most drugs. Thus, the dose-varying efficacy was only observed in a few drugs. Second, data on baseline characteristics (such as the proportion of patients using immunosuppressants) were not available in some trials. Therefore, the potential effect of these covariates may have been overlooked. Third, the exploration of covariates in our study was based on summary-level data and normalized to mean value. However, it should be noted that summary-level data may be associated with fallacies, so the results of covariate analysis of this study should be further confirmed by individual-level data.

## Conclusion

In conclusion, this study presented three separate models for clinical remission, clinical response, and endoscopic improvement in moderate-to-severe UC. Infliximab and upadacitinib were determined to be the most effective biologic and small targeted molecule, respectively. These models can be utilized to evaluate treatment efficacies in populations with varying characteristics. Younger patients with shorter UC duration, without prior anti-TNF treatment and current corticosteroids therapy, tended to show greater treatment effects. The findings may provide valuable insights for clinical practice.

## Data Availability

The original contributions presented in the study are included in the article/Supplementary Materials; further inquiries can be directed to the corresponding authors.

## Abbreviations

5-ASA: 5-aminosalicylates; CI: confidence interval; JAK: Janus kinase; MBMA: model-based meta-analysis; PRISMA: Preferred Reporting iItems for Systematic Reviews and Meta-Analyseis; S1PR: sphingosine-1-phosphate receptor; SLR: secondary loss of response; TNF: tumor necrosis factor; UC: ulcerative colitis; VPC: visual prediction check.
