# Supplementary material for: Quantitative evaluation of the time-course and efficacy of targeted agents for ulcerative colitis
Source: Front Pharmacol. 2024 Jun 5;15:1399963. doi: 10.3389/fphar.2024.1399963 (PMC11188356; doi:10.3389/fphar.2024.1399963)
Supplement: Supplementary file 1 [file DataSheet1.PDF]

Supplementary Materials

Quantitative Evaluation of Time-course and Efficacy of Targeted Agents for Ulcerative Colitis

Search Strategy

MEDLINE via PubMed

No	Search Details	Results
47	("randomized controlled trial"[Publication Type] OR "randomized"[Title/Abstract] OR "placebo"[Title/Abstract]) AND ("Infliximab"[Title/Abstract] OR "remicade"[Title/Abstract] OR "Infliximab"[MeSH Terms] OR ("Adalimumab"[Title/Abstract] OR "humira"[Title/Abstract] OR "Adalimumab"[MeSH Terms]) OR ("golimumab"[Title/Abstract] OR "CNTO-148"[Title/Abstract] OR "simponi"[Title/Abstract] OR "golimumab"[Supplementary Concept]) OR ("vedolizumab"[Title/Abstract] OR "MLN-0002"[Title/Abstract] OR "entyvio"[Title/Abstract] OR "vedolizumab"[Supplementary Concept]) OR ("etrolizumab"[Title/Abstract] OR "rhumab beta7"[Title/Abstract] OR "etrolizumab"[Supplementary Concept]) OR ("Ustekinumab"[Title/Abstract] OR "CNTO-1275"[Title/Abstract] OR "Ustekinumab"[MeSH Terms]) OR ("tofacitinib"[Title/Abstract] OR "CP-690550"[Title/Abstract] OR "xeljanz"[Title/Abstract] OR "tofacitinib"[Supplementary Concept]) OR ("filgotinib"[Title/Abstract] OR "GLPG0634"[Title/Abstract] OR "GLPG0634"[Supplementary Concept]) OR ("upadacitinib"[Title/Abstract] OR "ABT-494"[Title/Abstract] OR "upadacitinib"[Supplementary Concept]) OR ("ozanimod"[Title/Abstract] OR "RPC1063"[Title/Abstract] OR "ozanimod"[Supplementary Concept]) OR ("etrasimod"[Title/Abstract] OR "TD-1473"[Title/Abstract] OR "etrasimod"[Supplementary Concept]) OR "anti-TNF"[Title/Abstract] OR ("Janus Kinase Inhibitors"[MeSH Terms] OR "jak inhibitor"[Title/Abstract]) OR ("Sphingosine 1 Phosphate Receptor Modulators"[MeSH Terms] OR "sphingosine 1 phosphate receptor modulator"[Title/Abstract]) OR "anti-integrin"[Title/Abstract] OR ("antibod*"[Title/Abstract] OR "Antibodies"[MeSH Terms]) OR ("biologic*"[Title/Abstract] OR "Biological Products"[MeSH Terms]) OR "monoclonal*"[Title/Abstract]) AND (("ulcerative"[Title/Abstract] AND "colitis"[Title/Abstract]) OR "colitis, ulcerative"[MeSH Terms] OR "Inflammatory Bowel Diseases"[MeSH Terms] OR "inflammatory bowel disease*"[Title/Abstract] OR "IBD"[Title/Abstract])	1,619
46	"randomized controlled trial"[Publication Type] OR "randomized"[Title/Abstract] OR "placebo"[Title/Abstract]	972,568
45	"placebo"[Title/Abstract]	238,752

44	"randomized"[Title/Abstract]	629,696
43	"randomized controlled trial"[Publication Type]	579,215
42	"Infliximab"[Title/Abstract] OR "remicade"[Title/Abstract] OR "Infliximab"[MeSH Terms] OR "Adalimumab"[Title/Abstract] OR "humira"[Title/Abstract] OR "Adalimumab"[MeSH Terms] OR "golimumab"[Title/Abstract] OR "CNTO-148"[Title/Abstract] OR "simponi"[Title/Abstract] OR "golimumab"[Supplementary Concept] OR "vedolizumab"[Title/Abstract] OR "MLN-0002"[Title/Abstract] OR "entyvio"[Title/Abstract] OR "vedolizumab"[Supplementary Concept] OR "etrolizumab"[Title/Abstract] OR "rhumab beta7"[Title/Abstract] OR "etrolizumab"[Supplementary Concept] OR "Ustekinumab"[Title/Abstract] OR "CNTO-1275"[Title/Abstract] OR "Ustekinumab"[MeSH Terms] OR "tofacitinib"[Title/Abstract] OR "CP-690550"[Title/Abstract] OR "xeljanz"[Title/Abstract] OR "tofacitinib"[Supplementary Concept] OR "filgotinib"[Title/Abstract] OR "GLPG0634"[Title/Abstract] OR "GLPG0634"[Supplementary Concept] OR "upadacitinib"[Title/Abstract] OR "ABT-494"[Title/Abstract] OR "upadacitinib"[Supplementary Concept] OR "ozanimod"[Title/Abstract] OR "RPC1063"[Title/Abstract] OR "ozanimod"[Supplementary Concept] OR "etrasimod"[Title/Abstract] OR "TD-1473"[Title/Abstract] OR "etrasimod"[Supplementary Concept] OR "anti-TNF"[Title/Abstract] OR "Janus Kinase Inhibitors"[MeSH Terms] OR "jak inhibitor"[Title/Abstract] OR "Sphingosine 1 Phosphate Receptor Modulators"[MeSH Terms] OR "sphingosine 1 phosphate receptor modulator"[Title/Abstract] OR "anti-integrin"[Title/Abstract] OR "antibod*"[Title/Abstract] OR "Antibodies"[MeSH Terms] OR "biologic*"[Title/Abstract] OR "Biological Products"[MeSH Terms] OR "monoclonal*"[Title/Abstract]	2,810,844
41	"monoclonal*"[Title/Abstract]	240,458
40	"biologic*"[Title/Abstract] OR "Biological Products"[MeSH Terms]	1,643,391
39	"Biological Products"[MeSH Terms]	654,234
38	"antibod*"[Title/Abstract] OR "Antibodies"[MeSH Terms]	1,352,861
37	"Antibodies"[MeSH Terms]	893,967
36	"Sphingosine 1 Phosphate Receptor Modulators"[MeSH Terms] OR "sphingosine 1 phosphate receptor modulator"[Title/Abstract]	288
35	"Janus Kinase Inhibitors"[MeSH Terms] OR "jak inhibitor"[Title/Abstract]	2,381
34	"sphingosine 1 phosphate receptor modulator"[Title/Abstract]	172
33	"jak inhibitor"[Title/Abstract]	1,489
32	"anti-integrin"[Title/Abstract]	867
31	"Sphingosine 1 Phosphate Receptor Modulators"[MeSH Terms]	126
30	"Janus Kinase Inhibitors"[MeSH Terms]	1,206
29	"anti-TNF"[Title/Abstract]	12,279

28	"etrasimod"[Title/Abstract] OR "TD-1473"[Title/Abstract] OR "etrasimod"[Supplementary Concept]	26
27	"etrasimod"[Supplementary Concept]	5
26	"ozanimod"[Title/Abstract] OR "RPC1063"[Title/Abstract] OR "ozanimod"[Supplementary Concept]	183
25	"ozanimod"[Supplementary Concept]	75
24	"upadacitinib"[Title/Abstract] OR "ABT-494"[Title/Abstract] OR "upadacitinib"[Supplementary Concept]	395
23	"upadacitinib"[Supplementary Concept]	171
22	"filgotinib"[Title/Abstract] OR "GLPG0634"[Title/Abstract] OR "GLPG0634"[Supplementary Concept]	228
21	"GLPG0634"[Supplementary Concept]	95
20	"tofacitinib"[Title/Abstract] OR "CP-690550"[Title/Abstract] OR "xeljanz"[Title/Abstract] OR "tofacitinib"[Supplementary Concept]	2,468
19	"tofacitinib"[Supplementary Concept]	1,363
18	"Ustekinumab"[Title/Abstract] OR "CNTO-1275"[Title/Abstract] OR "Ustekinumab"[MeSH Terms]	2,851
17	"Ustekinumab"[MeSH Terms]	1,595
16	"etrolizumab"[Title/Abstract] OR "rhumab beta7"[Title/Abstract] OR "etrolizumab"[Supplementary Concept]	92
15	"etrolizumab"[Supplementary Concept]	41
14	"vedolizumab"[Title/Abstract] OR "MLN-0002"[Title/Abstract] OR "entyvio"[Title/Abstract] OR "vedolizumab"[Supplementary Concept]	1,587
13	"vedolizumab"[Supplementary Concept]	856
12	"golimumab"[Title/Abstract] OR "CNTO-148"[Title/Abstract] OR "simponi"[Title/Abstract] OR "golimumab"[Supplementary Concept]	1,541
11	"golimumab"[Supplementary Concept]	776
10	"Adalimumab"[Title/Abstract] OR "humira"[Title/Abstract] OR "Adalimumab"[MeSH Terms]	10,385
9	"Adalimumab"[MeSH Terms]	6,604
8	"Infliximab"[Title/Abstract] OR "remicade"[Title/Abstract] OR "Infliximab"[MeSH Terms]	17,024
7	"Infliximab"[MeSH Terms]	11,681
6	("ulcerative"[Title/Abstract] AND "colitis"[Title/Abstract]) OR "colitis, ulcerative"[MeSH Terms] OR "Inflammatory Bowel Diseases"[MeSH Terms] OR "inflammatory bowel disease*"[Title/Abstract] OR "IBD"[Title/Abstract]	125,367
5	"IBD"[Title/Abstract]	32,594
4	"inflammatory bowel disease*"[Title/Abstract]	60,386
3	"Inflammatory Bowel Diseases"[MeSH Terms]	93,336
2	"colitis, ulcerative"[MeSH Terms]	39,214
1	"ulcerative"[Title/Abstract] AND "colitis"[Title/Abstract]	47,627

Elsevier Embase

No.	Query	Results
#25	#3 AND #23 AND #24	7675
#24	'randomized controlled trial'/exp OR 'clinical trial'/exp	1911432
#23	#4 OR #5 OR #6 OR #7 OR #8 OR #9 OR #10 OR #11 OR #12 OR #13 OR #14 OR #15 OR #16 OR #17 OR #18 OR #19 OR #20 OR #21 OR #22	4209351
#22	'monoclonal antibody'/exp OR 'monoclonal antibody' OR monoclonal*:ab,kw,ti	923796
#21	'biological product'/exp OR biologic*:ab,kw,ti	2303333
#20	'antibody'/exp OR antibod*:ab,kw,ti	2131948
#19	'integrin inhibitor'/exp OR 'integrin inhibitor' OR 'anti-integrin':ab,kw,ti	1895
#18	'sphingosine 1 phosphate receptor modulator'/exp OR 'sphingosine 1 phosphate receptor modulator'	820
#17	'janus kinase inhibitor'/exp OR 'janus kinase inhibitor' OR 'jak inhibitor':ab,kw,ti	35807
#16	'tumor necrosis factor inhibitor'/exp OR 'tumor necrosis factor inhibitor'	130569
#15	'etrasimod'/exp OR 'etrasimod':ab,kw,ti OR 'td-1473':ab,kw,ti	282
#14	'ozanimod'/exp OR 'ozanimod':ab,kw,ti OR 'rpc1063':ab,kw,ti	1212
#13	'upadacitinib'/exp OR 'upadacitinib':kw,ti,ab OR 'abt-494':ab,kw,ti	3066
#12	'filgotinib'/exp OR 'filgotinib':ab,ti,kw OR 'glpg0634':ab,kw,ti	1417
#11	'tofacitinib'/exp OR 'cp-690550':ab,kw,ti OR 'xeljanz':ab,kw,ti OR 'jakvinus':ab,kw,ti OR 'tofacitinib':ab,kw,ti	11401
#10	'tofacitinib'/exp	11063
#9	'ustekinumab'/exp OR 'ustekinumab':kw,ti OR 'cnto-1275':ab,kw,ti	14083
#8	'etrolizumab'/exp OR 'pro145223':ab,kw,ti OR 'rhumab beta7':ab,kw,ti OR 'etrolizumab':ab,kw,ti	465
#7	'vedolizumab'/exp OR 'mln-0002':ab,kw,ti OR 'entyvio':ab,ti,kw OR 'vedolizumab':ab,kw,ti	9201
#6	'golimumab'/exp OR 'golimumab':ab,kw,ti OR 'cnto-148':ab,kw,ti OR 'simponi':ab,kw,ti	10867
#5	'adalimumab'/exp OR 'adalimumab':ab,kw,ti OR 'humira':ab,kw,ti	48932
#4	'infliximab'/exp OR 'infliximab':ab,kw,ti OR 'remicade':ab,kw,ti	66720
#3	#1 OR #2	242142
#2	'inflammatory bowel disease'/exp OR 'inflammatory bowel disease':ab,kw,ti	237789
#1	'ulcerative colitis'/exp OR 'ulcerative colitis':ab,ti,kw	113076

Wiley Cochrane

ID	Search	Hits
#1	MeSH descriptor: [Inflammatory Bowel Diseases] explode all trees	3770
#2	(inflammatory bowel disease*):ti,ab,kw (Word variations have been searched)	4189
#3	(IBD):ti,ab,kw (Word variations have been searched)	2171
#4	(ulcerative):ti,ab,kw (Word variations have been searched)	29485
#5	(colitis):ti,ab,kw (Word variations have been searched)	7127
#6	#4 and #5	5731
#7	#1 or #2 or #3 #6	6899
#8	MeSH descriptor: [Antibodies, Monoclonal] explode all trees	15926
#9	(infliximab):ti,ab,kw OR (remicade):ti,ab,kw (Word variations have been searched)	2541
#10	(adalimumab):ti,ab,kw OR (humira):ti,ab,kw (Word variations have been searched)	3654
#11	(golimumab):ti,ab,kw OR (CNTO-148):ti,ab,kw OR (simponi):ti,ab,kw (Word variations have been searched)	764
#12	(vedolizumab):ti,ab,kw OR (MLN-0002):ti,ab,kw OR (entyvio):ti,ab,kw (Word variations have been searched)	492
#13	(etrolizumab):ti,ab,kw OR (rhuMAb Beta7):ti,ab,kw (Word variations have been searched)	71
#14	(ustekinumab):ti,ab,kw OR (CNTO-1275):ti,ab,kw (Word variations have been searched)	1048
#15	(tofacitinib):ti,ab,kw OR (CP-690550):ti,ab,kw OR (xeljanz):ti,ab,kw OR (jakvinus):ti,ab,kw (Word variations have been searched)	990
#16	(filgotinib):ti,ab,kw OR (GLPG0634):ti,ab,kw (Word variations have been searched)	310
#17	(upadacitinib):ti,ab,kw OR (ABT-494):ti,ab,kw (Word variations have been searched)	558
#18	(ozanimod):ti,ab,kw OR (RPC1063):ti,ab,kw (Word variations have been searched)	168
#19	(etrasimod):ti,ab,kw OR (TD-1473):ti,ab,kw (Word variations have been searched)	72
#20	MeSH descriptor: [Janus Kinase Inhibitors] explode all trees	102
#21	(monoclonal*):ti,ab,kw (Word variations have been searched)	19470
#22	(biologic*):ti,ab,kw (Word variations have been searched)	38965
#23	(antibod*):ti,ab,kw (Word variations have been searched)	49260
#24	#8 or #9 or #10 or #11 or #12 or #13 or #14 or #15 or #16 or #17 or #18 or #19 or #20 or #21 or #22 or #23	94713
#25	#7 and #24	1936
trials 1896		

R code of the final models

R code of clinical remission model

```
cr99 <- function(trialno,arm,week,flag,dose,efficacy,no,
         male,white,age,weight,duration,leftside,extensive,
         Mayo,crp,TNF,corticoid,IS,smoker,
         ba,kp,A,eminf,emada,emgol,emved,emetr,emust,emmir,
         emtof,emfil,emupa,emoza,kada,kfil,koza,pduration,edur
)
{
  corp = 1
  corp = corp*((duration/mean(CLREM$duration))**pduration)
  pla = (ba + A*(1-exp(-exp(kp)*week)))*corp
  emtime = (1-exp(-exp(kada)*week))*(flag==3) +
    (1-exp(-exp(kfil)*week))*(flag==11) +
    (1-exp(-exp(koza)*week))*(flag==13) +
    1*(flag!=3&flag!=11&flag!=13)
  emrel = 1
  emrel = emrel*((duration/mean(CLREM$duration))**edur)
  emrel = emrel*emtime
  efinf = 0 + eminf*(flag==2)*(week!=0)
  efinf = efinf*emrel
  efada = 0 + emada*(flag==3)*(week!=0)
  efada = efada*emrel
  efgol = 0 + emgol*(flag==4)*(week!=0)
  efgol = efgol*emrel
  efved = 0 + emved*(flag==5)*(week!=0)
  efved = efved*emrel
  efetr = 0 + emetr*(flag==6)*(week!=0)
  efetr = efetr*emrel
  efust = 0 + emust*(flag==7)*(week!=0)
  efust = efust*emrel
  efmir = 0 + emmir*(flag==8)*(week!=0)
  efmir = efmir*emrel
  eftof = 0 + emtof*(flag==10)*(week!=0)
  eftof = eftof*emrel
  effil = 0 + emfil*(flag==11)*(week!=0)
  effil = effil*emrel
  efupa = 0 + emupa*(flag==12)*(week!=0)
  efupa = efupa*emrel
  efoza = 0 + emoza*(flag==13)*(week!=0)
  efoza = efoza*emrel
  emax = pla + efinf + efada + efgol + efved + efetr + efust + efmir +
    eftof + effil + efupa + efoza
  yp = 1/(1+exp(-emax))
```

```
  yp
} n99d <- gnls(efficacy~cr99(trialno,arm,week,flag,dose,efficacy,no,
                             male,white,age,weight,duration,leftside,extensive,
                             Mayo,crp,TNF,corticoid,IS,smoker,ba,kp,A,
                             eminf,emada,emgol,emved,emetr,emust,emmir,
                             emtof,emfil,emupa,emoza,kada,kfil,koza,pduration,edur
),
data = CLREM,
params = list(ba~1,kp~1,A~1,
              eminf~1,emada~1,emgol~1,emved~1,emetr~1,emust~1,emmir~1,
              emtof~1,emfil~1,emupa~1,emoza~1,kada~1,kfil~1,koza~1,pduration~1,
              edur~1
),
start = c(coef(n99)),
weights=varPower(0.5,form=~fitted(.)*(1-fitted(.))/no,fixed = 0.5),
correlation = corCompSymm(-0.3,form = ~tp1|group),
verbose = T
)
```

R code of clinical response model

```
cr39<- function(trialno,arm,week,flag,dose,corticoid,duration,efficacy,no,class,TNF,age,
                        pla,eminf,emada,emgol,emved,emetr,emust,emmir,
                        emtof,sfil,emupa,emoza,setra,kclass4,emupa45,ptnf,eage,ecor,edur
)
{
   prel = 0
   prel = prel + ((TNF-mean(CLRES$TNF))*ptnf)
   plac = pla + prel
   emrel = 0
   emrel = emrel+((age-mean(CLRES$age))*eage)
   emrel1 = 1
   emrel1 = emrel1*((corticoid/mean(CLRES$corticoid))**ecor)
   emrel1 = emrel1*((duration/mean(CLRES$duration))**edur)
   emtime = 1*(class!=4) +
      (1-exp(-exp(kclass4)*week))*(class==4)
   efinf = 0 + eminf*(flag==2)*(week!=0)
   efinf = (efinf*emrel1+emrel)*emtime
   efada = 0 + emada*(flag==3)*(week!=0)
   efada = (efada*emrel1+emrel)*emtime
   efgol = 0 + emgol*(flag==4)*(week!=0)
   efgol = (efgol*emrel1+emrel)*emtime
   efved = 0 + emved*(flag==5)*(week!=0)
   efved = (efved*emrel1+emrel)*emtime
   efetr = 0 + emetr*(flag==6)*(week!=0)
   efetr = (efetr*emrel1+emrel)*emtime
   efust = 0 + emust*(flag==7)*(week!=0)
   efust = (efust*emrel1+emrel)*emtime
   efmir = 0 + emmir*(flag==8)*(week!=0)
   efmir = (efmir*emrel1+emrel)*emtime
   eftof = 0 + emtof*(flag==10)*(week!=0)
   eftof = (eftof*emrel1+emrel)*emtime
   effil = 0 + sfil*dose*(flag==11)*(week!=0)
   effil = (effil*emrel1+emrel)*emtime
   efupa = 0 + emupa*(flag==12&dose!=45)*(week!=0) +
emupa45*(flag==12&dose==45)*(week!=0)
   efupa = (efupa*emrel1+emrel)*emtime
   efoza = 0 + emoza*(flag==13)*(week!=0)
   efoza = (efoza*emrel1+emrel)*emtime
   efetra = 0 + setra*dose*(flag==14)*(week!=0)
   efetra = (efetra*emrel1+emrel)*emtime
   emax = plac + efinf + efada + efgol + efved + efetr + efust + efmir +
      eftof + effil + efupa + efoza + efetra
   yp = 1/(1+exp(-emax))
```

```
   yp
}
n39d <- gnls(efficacy~cr39(trialno,arm,week,flag,dose,corticoid,duration,
                           efficacy,no,class,TNF,age,pla,
                           eminf,emada,emgol,emved,emetr,emust,emmir,
                           emtof,sfil,emupa,emoza,setra,kclass4,
                           emupa45,ptnf,eage,ecor,edur
),

data = CLRES,
params = list(eminf~1,emada~1,emgol~1,emved~1,emetr~1,emust~1,emmir~1,
              emtof~1,sfil~1,emupa~1,emoza~1,setra~1,kclass4~1,
              emupa45~1,ptnf~1,eage~1,ecor~1,edur~1
),
start = c(coef(n39)),
weights=varPower(0.5,form=~fitted(.)*(1-fitted(.))/no,fixed = 0.5),
correlation = corCompSymm(form = ~tp1|group),
verbose = T
)
```

R code of endoscopic improvement model

```
cr42 <- function(trialno,arm,week,flag,dose,group,tp1,
                    efficacy,no,corticoid,TNF,Mayo,duration,
                    pla,eminf,emada,emgol,emved,emetr,emust,emmir,
                    emtof,emfil,supa,emoza,setra,etnf,edur,pdur
)
{
  prel = 1
  prel = prel*((duration/mean(ENIMP$duration))**pdur)
  plac = pla
  plac = plac*prel
  emrel = 0
  emrel = emrel + ((TNF-mean(ENIMP$TNF))*etnf)
  emrel = emrel + ((duration-mean(ENIMP$duration))*edur)
  emtime = 1
  emrel = emrel*emtime
  efinf = 0 + eminf*(flag==2)*(week!=0)
  efinf = efinf+emrel
  efada = 0 + emada*(flag==3)*(week!=0)
  efada = efada+emrel
  efgol = 0 + emgol*(flag==4)*(week!=0)
  efgol = efgol+emrel
  efved = 0 + emved*(flag==5)*(week!=0)
  efved = efved+emrel
  efetr = 0 + emetr*(flag==6)*(week!=0)
  efetr = efetr+emrel
  efust = 0 + emust*(flag==7)*(week!=0)
  efust = efust+emrel
  efmir = 0 + emmir*(flag==8)*(week!=0)
  efmir = efmir+emrel
  eftof = 0 + emtof*(flag==10)*(week!=0)
  eftof = eftof+emrel
  effil = 0 + emfil*(flag==11)*(week!=0)
  effil = effil+emrel
  efupa = 0 + (supa*dose)*(flag==12)*(week!=0)
  efupa = efupa+emrel
  efoza = 0 + emoza*(flag==13)*(week!=0)
  efoza = efoza+emrel
  efetra = 0 + (setra*dose)*(flag==14)*(week!=0)
  efetra = efetra+emrel
  emax = plac + efinf + efada + efgol + efved + efetr + efust + efmir +
    eftof + effil + efupa + efoza + efetra
  yp = 1/(1+exp(-emax))
  yp
```

```
}
n42d <- gnls(efficacy~cr42(trialno,arm,week,flag,dose,group,tp1,
                           efficacy,no,corticoid,TNF,Mayo,duration,pla,
                           eminf,emada,emgol,emved,emetr,emust,emmir,
                           emtof,emfil,supa,emoza,setra,etnf,edur,pdur
),

data = ENIMP,
params = list(eminf~1,emada~1,emgol~1,emved~1,emetr~1,emust~1,emmir~1,
              emtof~1,emfil~1,supa~1,emoza~1,setra~1,etnf~1,edur~1,pdur~1
),
start = coef(n42),
weights=varPower(0.5,form=~fitted(.)*(1-fitted(.))/no,fixed = 0.5),
correlation = corCompSymm(form = ~tp1|group),
verbose = T
)
```

Table S1 Summary of included studies and reported time points

No.	Study	Patients	Drug	Regimen	Time points (week)		
					Clinical remission	Clinical response	Endoscopic improvement
1	Rutgeerts, 2005 (ACT1)[1]	364	infliximab	5 mg/kg 0, 2, 6, Q8W	8, 30, 54	8, 30, 54	8, 30, 54
				10 mg/kg 0, 2, 6, Q8W			
2	Rutgeerts, 2005 (ACT2)[1]	364	infliximab	5 mg/kg 0, 2, 6, Q8W	8, 30	8, 30	8, 30
				10 mg/kg 0, 2, 6, Q8W			
3	Xian-Janssen, 2014 (REMICADEUCO3001)[2]	99	infliximab	5 mg/kg 0, 2, 6, Q8W	8, 26	8, 26	8, 26
4	Panaccione, 2014 (UC SUCCESS)[3]	239	infliximab	5 mg/kg 0, 2, 6, Q8W	NA	NA	16
5	Jiang, 2015[4]	123	infliximab	3.5 mg/kg 0, 2, 6, Q8W	8, 30	8, 30	8, 30
				5 mg/kg 0, 2, 6, Q8W			
6	Kobayashi, 2016 (Japic CTI-060298)[5]	208	infliximab	5 mg/kg 0, 2, 6, Q8W	8, 30	8, 30	8, 30
7	Reinisch, 2011 (ULTRA1)[6]	576	adalimumab	160 mg 0w+80 mg 2w+40 mg EOW	8	8	8
				80 mg 0w+40 mg 2w+40 mg EOW			
8	Sandborn, 2012 (ULTRA2)[7]	518	adalimumab	160 mg 0w+80 mg 2w+40 mg EOW	8, 52	8, 52	8, 52

No.	Study	Patients	Drug	Regimen	Time points (week)		
					Clinical remission	Clinical response	Endoscopic improvement
9	Suzuki, 2014 (NCT00853099)[8]	274	adalimumab	160 mg 0w+80 mg 2w+40 mg EOW	8, 32, 52	2, 4, 6, 8, 32, 52	8, 32, 52
				80 mg 0w+40 mg 2w+40 mg EOW			
10	Colombel, 2019 (SERENE-UC)[9]	952	adalimumab	160 mg 0w+80 mg 2w+40 mg EOW	8	8	8
				160 mg 0, 1,2, 3w+40 mg 4w+40 mg EOW			
11	Sandborn, 2014 (PURSUIT SC)[10]	1065	golimumab	100 mg 0w+50 mg 2w	6	6	6
				200mg 0w+100 mg 2w			
				400mg 0w+200 mg 2w			
12	Feagan, 2013 (GEMINI1)[11]	895	vedolizumab	300 mg 0, 2W	6	6	6
13	Sands, 2019 (VARSITY)[12]	1285	adalimumab	160 mg 0w+80 mg 2w+40 mg EOW	14, 52	2, 4, 6, 14, 22, 30, 38, 46, 52	52
			vedolizumab	300 mg 0, 2, 6, Q8W			
14	Motoya, 2019 (NCT02039505)[13]	292	vedolizumab	300 mg 0, 2, 6, Q8W	10	10	10
15	Vermeire, 2014 (EUCALYPTUS)[14]	124	etrolizumab	100 mg 0, 4, 8W	6, 10	6, 10	6, 10
				420 mg 0W+300 mg 2, 4, 8W			

No.	Study	Patients	Drug	Regimen	Time points (week)		
					Clinical remission	Clinical response	Endoscopic improvement
16	Rubin, 2021 (HIBISCUS1)[15]	358	adalimumab	160 mg 0w+80 mg 2w+40 mg EOW	10	10	10
			etrolizumab	105 mg Q4W			
17	Rubin, 2021 (HIBISCUS2)[15]	358	adalimumab	160 mg 0w+80 mg 2w+40 mg EOW	10	10	10
			etrolizumab	105 mg Q4W			
18	Peyrin-Biroulet, 2021 (HICKORY)[16]	609	etrolizumab	105 mg Q4W	14	14	14
19	Danese, 2021 (GARDENIA)[17]	397	infliximab	5 mg/kg 0, 2, 6, Q8W	10, 54	10	10, 54
			etrolizumab	105 mg Q4W			
20	Sands, 2019 (UNIFI)[18]	961	ustekinumab	130 mg	8	8	8
				6 mg/kg			
21	Sandborn, 2012 (NCT00787202)[19]	195	tofacitinib	0.5 mg BID	8	8	NA
				3 mg BID			
				10 mg BID			
				15 mg BID			
22	Sandborn, 2017 (OCTAVE1)[20]	614	tofacitinib	10 mg BID	8	8	8
				15 mg BID			
23	Sandborn, 2017 (OCTAVE2)[20]	614	tofacitinib	10 mg BID	8	8	8
				15 mg BID			
24	Feagan, 2021 (SELECTION-1)[21]	1090	filgotinib	100 mg QD	10, 58	10, 58	10, 58
				200 mg QD			

No.	Study	Patients	Drug	Regimen	Time points (week)		
					Clinical remission	Clinical response	Endoscopic improvement
25	Feagan, 2021 (SELECTION-2)[21]	950	filgotinib	100 mg QD	10,58	10,58	10,58
				200 mg QD			
27	Sandborn, 2016 (TOUCHSTONE)[22]	199	ozanimod	0.5 mg QD	NA	10	8, 32
				1 mg QD			
26	Sandborn, 2021 (TRUE NORTH)[23]	1012	ozanimod	1 mg QD	8. 32	8, 32	10
30	Sandborn, 2020 (U-ACHIEVE-Substudy1)[24]	250	upadacitinib	7.5 mg QD	8	8	8
				15 mg QD			
				30 mg QD			
				45 mg QD			
28	Danese, 2022 (U-ACHIEVE-Substudy2)[25]	474	upadacitinib	45 mg QD	8	8	8
29	Danese, 2022 (U-ACCOMPLISH)[25]	522	upadacitinib	45 mg QD	8	8	8
31	Sandborn, 2020 (NCT02589665)[26]	358	mirikizumab	50 mg Q4W	12	12	12
				200 mg Q4W			
				600 mg Q4W			
32	D' Haens, 2022 (LUCENT 1)[27]	1281	mirikizumab	300 mg Q4W	12	12	12
33	Sandborn, 2019 (OASIS)[28]	156	etrasimod	1 mg QD	NA	12	12
				2 mg QD			

No.	Study	Patients	Drug	Regimen	Time points (week)		
					Clinical remission	Clinical response	Endoscopic improvement
34	Sandborn, 2022 (ELEVATE UC 52)[29]	433	etrasimod	2 mg QD	NA	12, 52	12, 52
35	Sandborn, 2022 (ELEVATE UC 12)[29]	354	etrasimod	2 mg QD	NA	12	12

NA, not available.

List of included studies:

1 Rutgeerts P, Sandborn WJ, Feagan BG, et al. Infliximab for induction and maintenance therapy for ulcerative colitis. *The New England journal of medicine.* 2005;**353(23):**2462-2476.

2 A Study to Evaluate the Effectiveness and Safety of Infliximab in Chinese Patients With Active Ulcerative Colitis. 2015. (Available from:) https://clinicaltrials.gov/study/NCT01551290.

3 Panaccione R, Ghosh S, Middleton S, et al. Combination therapy with infliximab and azathioprine is superior to monotherapy with either agent in ulcerative colitis. *Gastroenterology.* 2014;**146(2)**.

4 Jiang X-L, Cui H-F, Gao J, Fan H. Low-dose Infliximab for Induction and Maintenance Treatment in Chinese Patients With Moderate to Severe Active Ulcerative Colitis. *Journal of Clinical Gastroenterology.* 2015;**49(7):**582-588.

5 Kobayashi T, Suzuki Y, Motoya S, et al. First trough level of infliximab at week 2 predicts future outcomes of induction therapy in ulcerative colitis-results from a multicenter prospective randomized controlled trial and its post hoc analysis. *Journal of Gastroenterology.* 2016;**51(3):**241-251.

6. Reinisch W, Sandborn WJ, Hommes DW, et al. Adalimumab for induction of clinical remission in moderately to severely active ulcerative colitis: results of a randomised controlled trial. *Gut.* 2011;**60(6):**780-787.

7 Sandborn WJ, van Assche G, Reinisch W, et al. Adalimumab induces and maintains clinical remission in patients with moderate-to-severe ulcerative colitis. *Gastroenterology.* 2012;**142(2)**.

8 Suzuki Y, Motoya S, Hanai H, et al. Efficacy and safety of adalimumab in Japanese patients with moderately to severely active ulcerative colitis. *Journal of Gastroenterology.* 2014;**49(2)**:283-294.

9 High versus standard adalimumab induction dosing regimens in patients with moderately to severely active ulcerative colitis: results from the SERENE-UC induction study. United European Gastroenterol J 2019;**7 (Suppl):**118.

10 Sandborn WJ, Feagan BG, Marano C, et al. Subcutaneous golimumab induces clinical response and remission in patients with moderate-to-severe ulcerative colitis. *Gastroenterology.* 2014;**146(1).**

11 Feagan BG, Rutgeerts P, Sands BE, et al. Vedolizumab as induction and maintenance therapy for ulcerative colitis. *The New England journal of medicine.* 2013;**369(8):**699-710.

12 Sands BE, Peyrin-Biroulet L, Loftus EV, et al. Vedolizumab versus Adalimumab for Moderate-to-Severe Ulcerative Colitis. *The New England journal of medicine.* 2019;**381(13):**1215-1226.

13 Motoya S, Watanabe K, Ogata H, et al. Vedolizumab in Japanese patients with ulcerative colitis: A Phase 3, randomized, double-blind, placebo-controlled study. *PloS One.* 2019;**14(2):**e0212989.

14 Vermeire S, O'Byrne S, Keir M, et al. Etrolizumab as induction therapy for ulcerative colitis: a randomised, controlled, phase 2 trial. *Lancet (London, England).* 2014;**384(9940):**309-318.

15 Rubin DT, Dotan I, DuVall A, et al. Etrolizumab versus adalimumab or placebo as induction therapy for moderately to severely active ulcerative colitis (HIBISCUS): two phase 3 randomised, controlled trials. *The Lancet Gastroenterology & Hepatology.* 2022;**7(1):**17-27.

16 Peyrin-Biroulet L, Hart A, Bossuyt P, et al. Etrolizumab as induction and maintenance therapy for ulcerative colitis in patients previously treated with tumour necrosis factor inhibitors (HICKORY): a phase 3, randomised, controlled trial. *The Lancet Gastroenterology & Hepatology.* 2022;**7(2):**128-140.

17 Danese S, Colombel J-F, Lukas M, et al. Etrolizumab versus infliximab for the treatment of moderately to severely active ulcerative colitis (GARDENIA): a randomised, double-blind, double-dummy, phase 3 study. *The Lancet Gastroenterology & Hepatology.* 2022;**7(2):**118-127.

18 Sands BE, Sandborn WJ, Panaccione R, et al. Ustekinumab as Induction and Maintenance Therapy for Ulcerative Colitis. *The New England journal of medicine.* 2019;**381(13):**1201-1214.

19 Sandborn WJ, Ghosh S, Panes J, et al. Tofacitinib, an oral Janus kinase inhibitor, in active ulcerative colitis. *The New England journal of medicine.* 2012;**367(7):**616-624.

20 Sandborn WJ, Su C, Sands BE, et al. Tofacitinib as Induction and Maintenance Therapy for Ulcerative Colitis. *The New England journal of medicine.* 2017;**376(18):**1723-1736.

21 Feagan BG, Danese S, Loftus EV, et al. Filgotinib as induction and maintenance therapy for ulcerative colitis (SELECTION): a phase 2b/3 double-blind, randomised, placebo-controlled trial. *Lancet (London, England).* 2021;**397(10292):**2372-2384.

22 Sandborn WJ, Feagan BG, Wolf DC, et al. Ozanimod Induction and Maintenance Treatment for Ulcerative Colitis. *The New England journal of medicine.*

2016;**374(18):**1754-1762.
23 Sandborn WJ, Feagan BG, D'Haens G, et al. Ozanimod as Induction and Maintenance Therapy for Ulcerative Colitis. *The New England journal of medicine.* 2021;**385(14):**1280-1291.
24 Sandborn WJ, Ghosh S, Panes J, et al. Efficacy of Upadacitinib in a Randomized Trial of Patients With Active Ulcerative Colitis. *Gastroenterology.* 2020;**158(8)**.
25 Danese S, Vermeire S, Zhou W, et al. Upadacitinib as induction and maintenance therapy for moderately to severely active ulcerative colitis: results from three phase 3, multicentre, double-blind, randomised trials. *Lancet (London, England).* 2022;**399(10341):**2113-2128.
26 Sandborn WJ, Ferrante M, Bhandari BR, et al. Efficacy and Safety of Mirikizumab in a Randomized Phase 2 Study of Patients With Ulcerative Colitis. *Gastroenterology.* 2020;**158(3)**.
27 Efficacy and Safety of Mirikizumab as Induction Therapy in Patients With Moderately to Severely Active Ulcerative Colitis: Results From the Phase 3 LUCENT-1 Study. *Gastroenterology & Hepatology.* 2022;**18(4 Suppl 1):**7-8.
28 Sandborn WJ, Peyrin-Biroulet L, Zhang J, et al. Efficacy and Safety of Etrasimod in a Phase 2 Randomized Trial of Patients With Ulcerative Colitis. *Gastroenterology.* 2020;**158(3):**550-561.
29 Sandborn WJ, Vermeire S, Peyrin-Biroulet L, et al. Etrasimod as induction and maintenance therapy for ulcerative colitis (ELEVATE): two randomised, double-blind, placebo-controlled, phase 3 studies. *Lancet (London, England).* 2023;**401(10383):**1159-1171.

Figure S1 Flow diagram for study selection

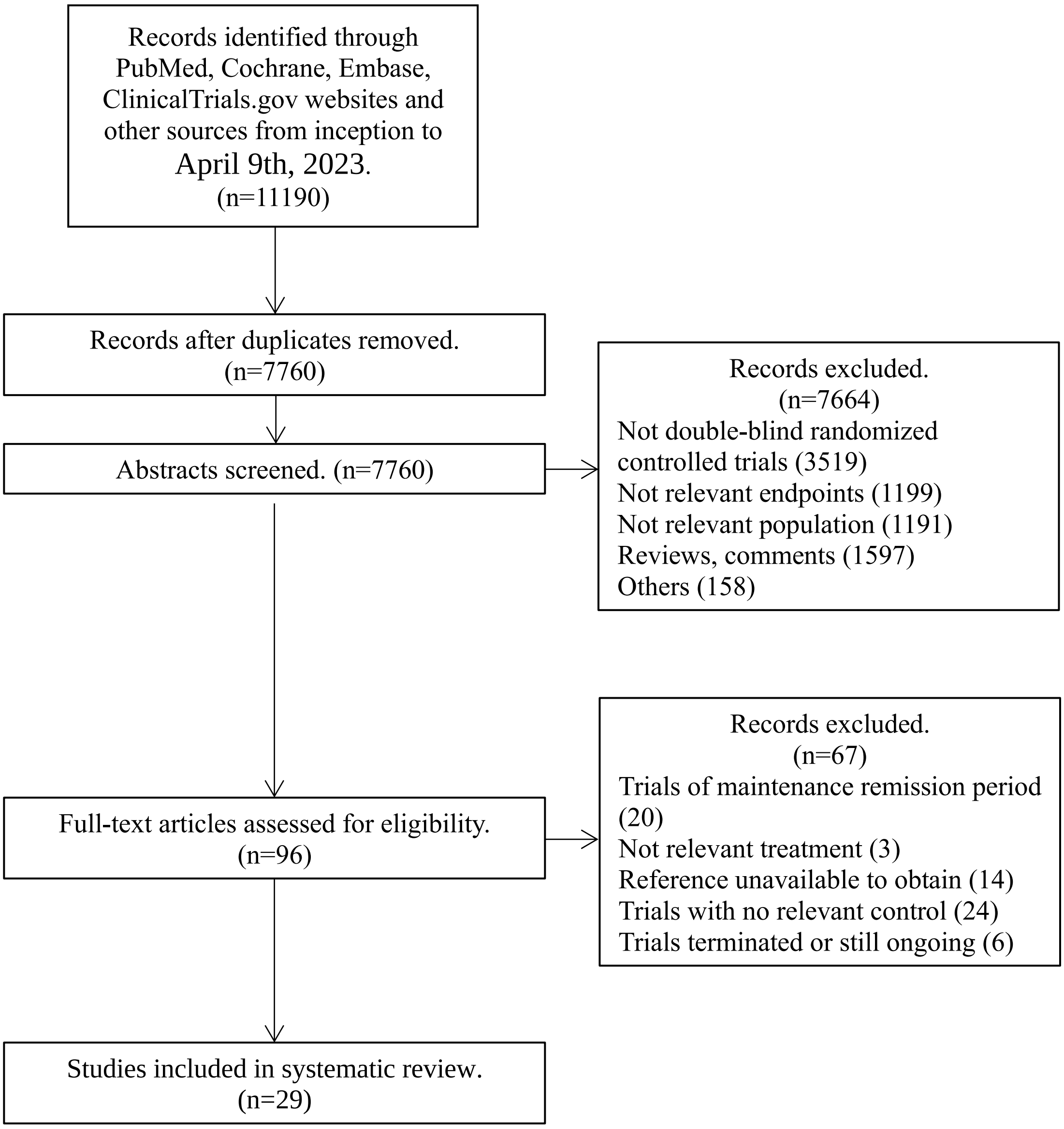

Figure S2 Summary of study-level literature assessment results

Study	Random sequence generation (selection bias)	Allocation concealment (selection bias)	Blinding of participants and personnel (performance bias)	Blinding of outcome assessment (detection bias)	Incomplete outcome data (attrition bias)	Selective reporting (reporting bias)	Other bias
Colombel, 2019 (SERENE-UC)	+	+	+	+	+	+	+
D' Haens, 2022 (LUCENT 1)	+	+	+	+	+	+	+
Danese, 2021 (GARDENIA)	+	+	+	+	+	+	+
Danese, 2022 (U-ACCOMPLISH)	+	+	+	+	+	+	+
Danese, 2022 (U-ACHIEVE-Substudy2)	+	+	+	+	+	+	+
Feagan, 2013 (GEMINI1)	+	+	+	+	+	+	+
Feagan, 2021 (SELECTION-1)	+	+	+	+	+	+	+
Feagan, 2021 (SELECTION-2)	+	+	+	+	+	+	+
Jiang, 2015	+	?	+	+	+	+	+
Kobayashi, 2016 (Japic CTI-060298)	+	+	+	+	+	+	+
Motoya, 2019 (NCT02039505)	+	?	+	+	+	+	+
Panaccione, 2014 (UC SUCCESS)	+	+	+	+	+	+	+
Peyrin-Biroulet, 2021 (HICKORY)	+	+	+	+	+	+	+
Reinisch, 2011 (ULTRA1)	+	+	+	+	+	+	+
Rubin, 2021 (HIBISCUS1)	+	+	+	+	+	+	+
Rubin, 2021 (HIBISCUS2)	+	+	+	+	+	+	+
Rutgeerts, 2005 (ACT1)	+	+	+	+	+	+	+
Rutgeerts, 2005 (ACT2)	+	+	+	+	+	+	+
Sandborn, 2012 (NCT00787202)	+	+	+	+	+	+	+
Sandborn, 2012 (ULTRA2)	+	+	+	+	+	+	+
Sandborn, 2014 (PURSUIT SC)	+	?	+	+	+	+	+
Sandborn, 2016 (TOUCHSTONE)	+	+	+	+	+	+	+
Sandborn, 2017 (OCTAVE1)	+	?	+	+	+	+	+
Sandborn, 2017 (OCTAVE2)	+	?	+	+	+	+	+
Sandborn, 2019 (OASIS)	+	+	+	+	+	+	+
Sandborn, 2020 (NCT02589665)	+	+	+	+	+	+	+
Sandborn, 2020 (U-ACHIEVE-Substudy1)	+	+	+	+	+	+	+
Sandborn, 2021 (TRUE NORTH)	+	+	+	+	+	+	+
Sandborn, 2022 (ELEVATE UC 12)	+	+	+	+	?	?	+
Sandborn, 2022 (ELEVATE UC 52)	+	+	+	+	?	?	+
Sands, 2019 (UNIFI)	+	+	+	+	+	+	+
Sands, 2019 (VARSITY)	+	+	+	+	+	+	+
Suzuki, 2014 (NCT00853099)	+	+	+	+	+	+	+
Vermeire, 2014 (EUCALYPTUS)	+	+	+	+	+	+	+
Xian-Janssen , 2014 (REMICADEUCO3001)	?	+	+	+	+	+	+

Figure S3 Summary of literature assessment results

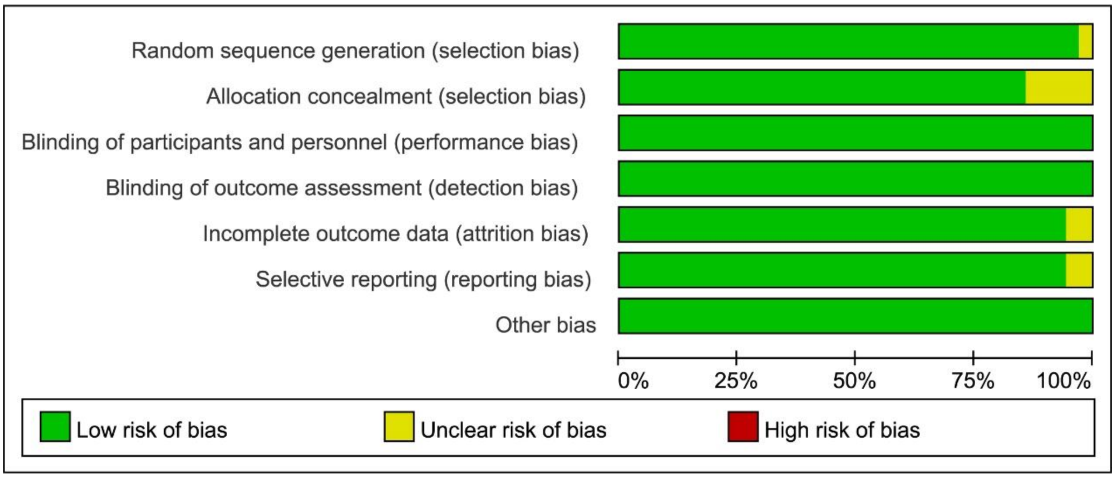

Figure S4 The model diagnostic plots

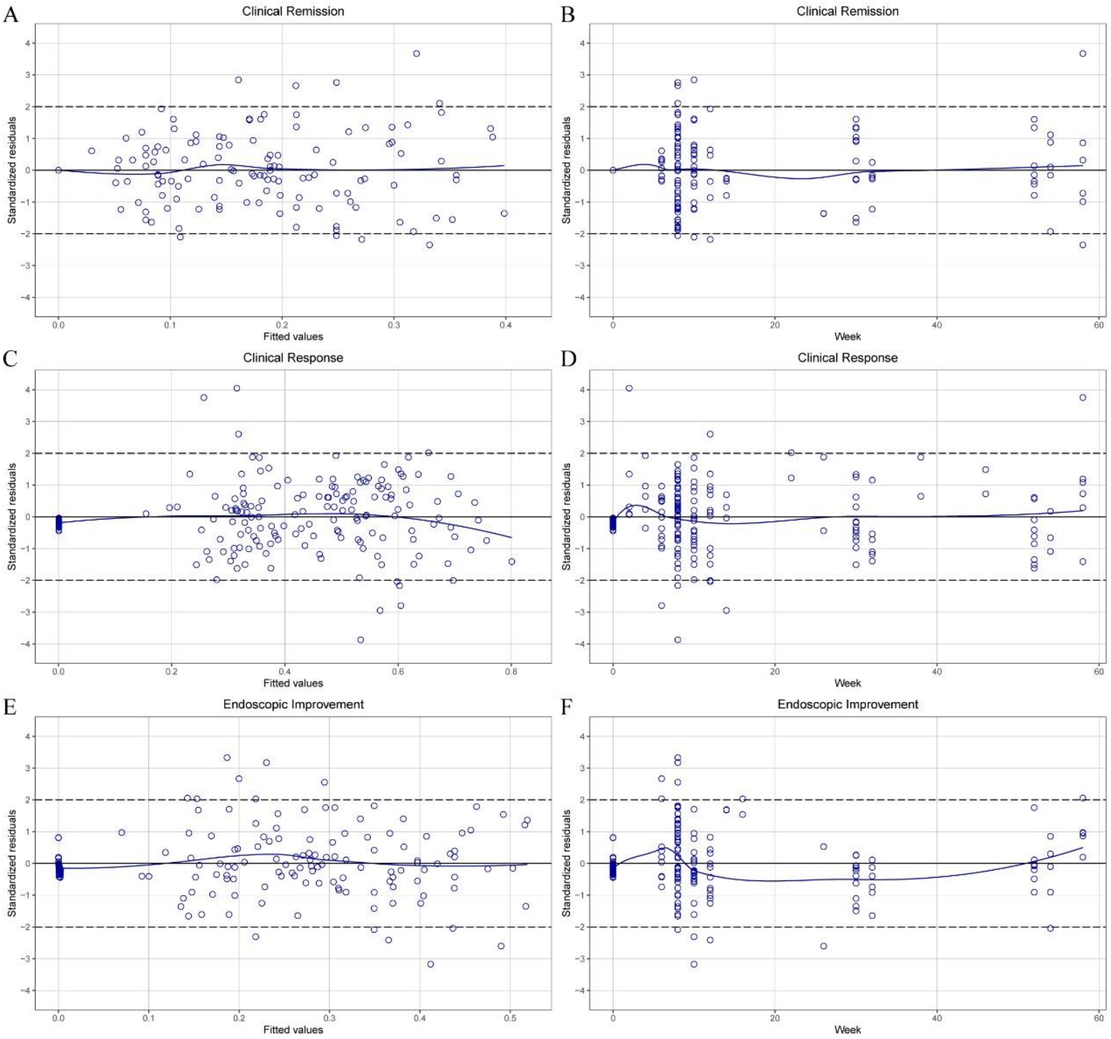

Model diagnostic plots of clinical remission model (A and B), clinical response model (C and D) and endoscopic improvement (E and F). The blue solid lines represent the regression lines. The blue and black dashed lined are the position where standardized residuals equal to 0 and ± 2.

Figure S5 Model fitted time-course plots

(A)

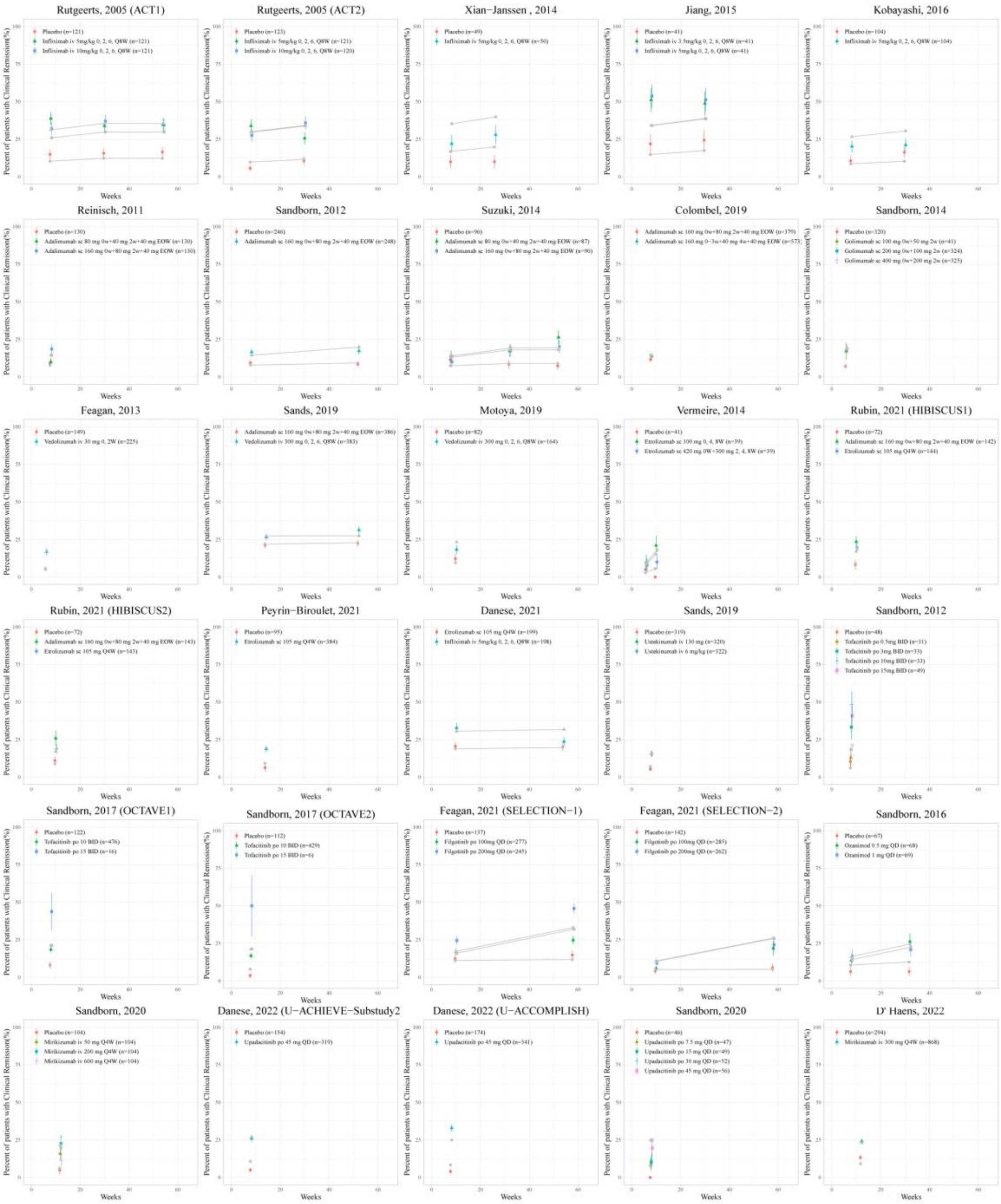

(B)

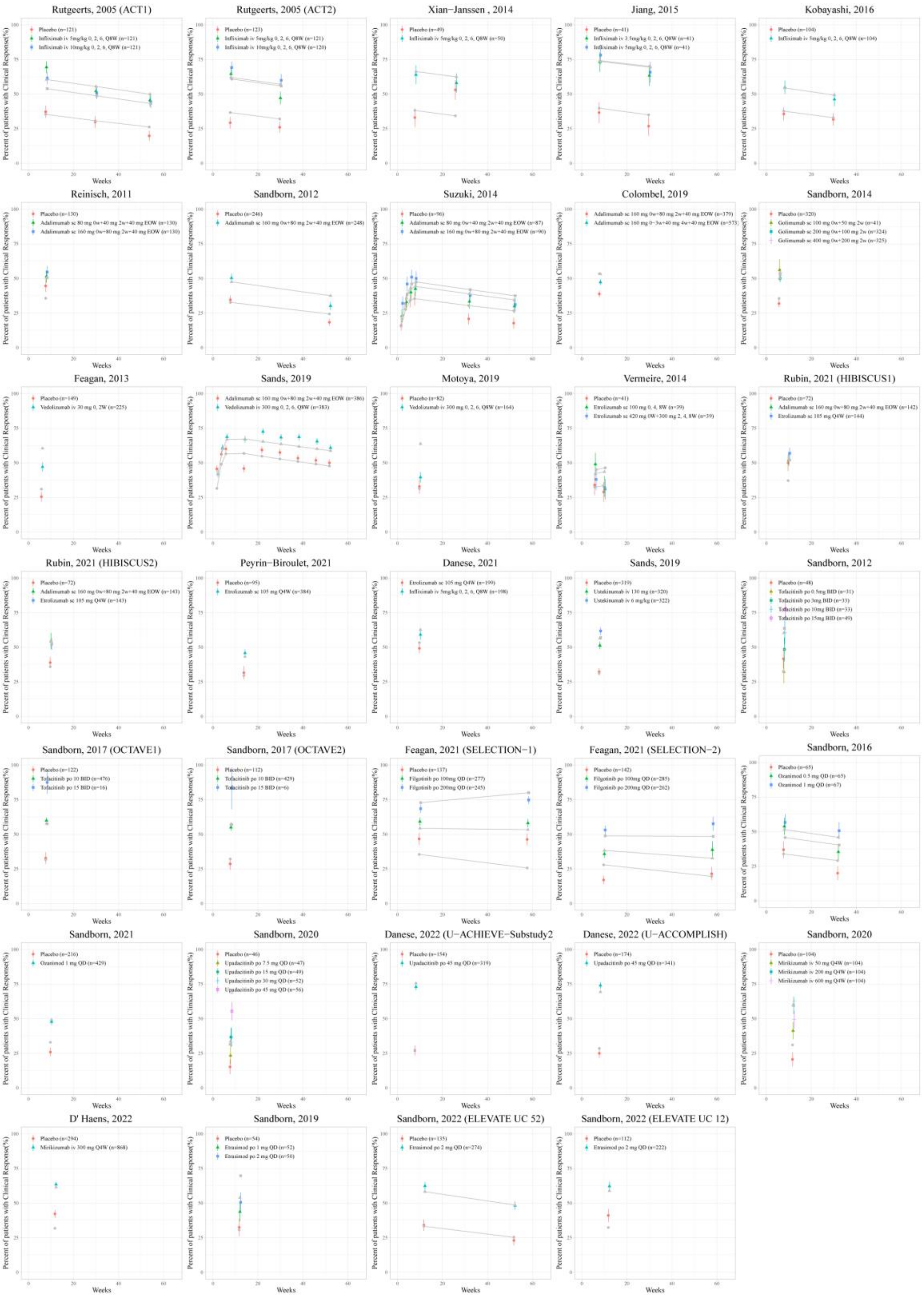
Rutgeerts, 2005 (ACT1)
Rutgeerts, 2005 (ACT2)
Xian−Janssen , 2014
Jiang, 2015
Kobayashi, 2016
Reinisch, 2011
Sandborn, 2012
Suzuki, 2014
Colombel, 2019
Sandborn, 2014
Feagan, 2013
Sands, 2019
Motoya, 2019
Vermeire, 2014
Rubin, 2021 (HIBISCUS1)
Rubin, 2021 (HIBISCUS2)
Peyrin−Biroulet, 2021
Danese, 2021
Sands, 2019
Sandborn, 2012
Sandborn, 2017 (OCTAVE1)
Sandborn, 2017 (OCTAVE2)
Feagan, 2021 (SELECTION−1)
Feagan, 2021 (SELECTION−2)
Sandborn, 2016
Sandborn, 2021
Sandborn, 2020
Danese, 2022 (U−ACHIEVE−Substudy2
Danese, 2022 (U−ACCOMPLISH)
Sandborn, 2020
D' Haens, 2022
Sandborn, 2019
Sandborn, 2022 (ELEVATE UC 52)
Sandborn, 2022 (ELEVATE UC 12)
Percent of patients with Clinical Response(%)
Weeks

(C)

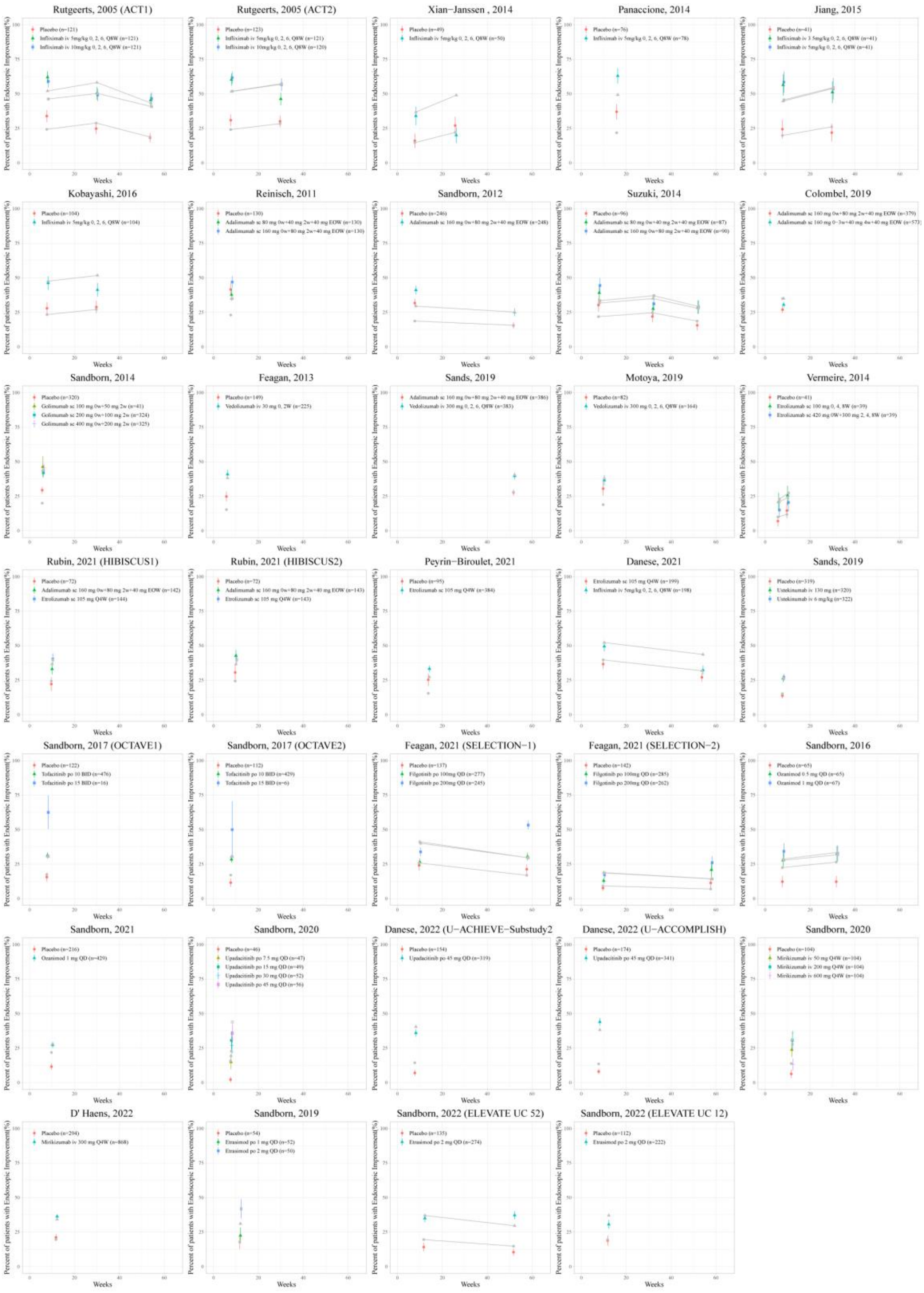

Model fitted time-course plots of clinical remission (A), clinical response (B), and endoscopic improvement (C). Color symbols and vertical bars are observed mean and calculated weight of time points; gray symbols and lines are the model predictions. QD, once daily; BID, twice daily; q2w, once every other week; q4w, once every 4 weeks.
